# Proinsulin regulators identified with CRISPR screen and in vivo mouse QTL mapping

**DOI:** 10.1038/s41467-026-71726-z

**Published:** 2026-04-13

**Authors:** Sisi Lai, Mark P. Keller, Jinglin Zhang, Zhou Fang, Ying Xie, Chen Weng, Saixian Zhang, Shanshan Zhang, Peidong Gao, Luxin Ke, Yuntong Wang, Kelly A. Mitok, Lauren Clark, Kathryn L. Schueler, Hanxiao Liu, Betul Hatipoglu, Maria Hatzoglou, Yuanyuan Chen, Anath Shalev, Fulai Jin, Alan D. Attie, Yan Li

**Affiliations:** 1 Department of Genetics and Genome Sciences, Case Western Reserve University, Cleveland, OH USA; 2 The Biomedical Sciences Training Program (BSTP), School of Medicine, Case Western Reserve University, Cleveland, OH USA; 3 Department of Biochemistry, University of Wisconsin-Madison, Madison, WI USA; 4 Case Western Reserve University School of Medicine, Cleveland, OH USA; 5 Department of Medicine and Department of Endocrinology, University Hospitals Cleveland Medical Center, Cleveland, OH USA; 6 Department of Ophthalmology and Department of Pharmacology and Chemical Biology, University of Pittsburgh, Pittsburgh, PA USA; 7 Comprehensive Diabetes Center, Division of Endocrinology, Diabetes and Metabolism, Department of Medicine, University of Alabama at Birmingham, Birmingham, AL USA; 8 Department of Computer and Data Sciences and Department of Population and Quantitative Health Sciences, Case Western Reserve University, Cleveland, OH USA; 9 Case Comprehensive Cancer Center, Case Western Reserve University, Cleveland, OH USA

**Keywords:** Functional genomics, Diabetes

## Abstract

Altered proinsulin levels in β-cells and bloodstream are hallmarks of diabetes and other diseases, but our knowledge about the proinsulin regulators remains limited. Here we perform a genome-wide CRISPR screen to identify 84 proinsulin regulators that alter intracellular proinsulin/insulin ratio in a mouse β-cell line. The proinsulin regulators are distinct from the insulin regulators from a previous orthogonal CRISPR screen. Functional annotation of the proinsulin regulators highlights Golgi as the primary organelle for proinsulin storage and regulation. Trafficking towards the Golgi increases the intra-cellular proinsulin/insulin ratio, while trafficking away from the Golgi, including exocytosis and Golgi-to-ER retrograde transport, decreases the intracellular proinsulin levels. We also map mouse quantitative trait loci (QTLs) associated with plasma proinsulin levels and use the CRISPR screen results to pinpoint the causal genes within the QTL loci. Interestingly, protein disulfide isomerase *Pdia6* is the strongest hit from both CRISPR screen and the in vivo QTL mapping. Knocking down *Pdia6* significantly reduce proinsulin accumulation in Golgi and secretory granules. Intriguingly, *Pdia6*-depletion in both human and mouse β-cells does not affect the folding status of proinsulin but causes significantly impaired proinsulin production through a UPR-independent mechanism. Taken together, our genetic profiles provide mechanistic insights into the regulation of proinsulin/insulin homeostasis.

## Abstract (web summary)

This study uses genome-wide screening and mouse genetics to uncover how cells control proinsulin, identifying the Golgi as a central hub and revealing PDIA6 as a key regulator of proinsulin production in pancreatic β-cells.

## Introduction

Insulin is produced in pancreatic β-cells through a tightly orchestrated multi-step process^1–3^. *INS* gene transcription is controlled by key β-cell transcription factors such as *PDX1*, *NEUROD1*, and *MAFA*^4–10^. After pre-proinsulin enters the ER through translocon-mediated localization, it forms disulfide bonds in the ER oxidative environment before being transported through the Golgi apparatus. Proinsulin is then sorted into the immature granules, where they are proteolytically cleaved by enzymes including *PCSK1*, *PCSK2*, and *CPE*. Mature insulin is then stored in mature granules as an anhydrous crystal bound to Zn^2+^^2,11,12^.

Pancreatic β-cells have a heavy burden of insulin processing and production since every β-cell can synthesize up to 6000 proinsulin/insulin molecules per second. Upon glucose stimulation, insulin synthesis represents 30–50% of total protein synthesis^13–18^. Dysregulation of insulin processing can cause diabetes. For example, substitution of proinsulin cysteine residues interferes with disulfide bond formation directly causes insulin deficiency and Mutant INS-gene-induced Diabetes of Youth (MIDY)^12,19,20^. Mutations in *PCSK1* and *PCSK2* also cause obesity, glucose intolerance, and increased risk of type 2 diabetes (T2D)^21–23^.

A small fraction of proinsulin (~3%)^24^ may escape the conversion process and be stored together with insulin in the mature granules, which upon stimulation, will be co-released into the bloodstream. Although proinsulin is far less active in stimulating glucose uptake and inhibiting hepatic glucose output^25^, elevated circulating proinsulin and an increased proinsulin-to-insulin ratio are found in a wide range of diseases, including obesity, insulinoma, type 1 diabetes (T1D), and T2D^26–34^. This abnormality is even observed at the prediabetic stage among high-risk T1D individuals (relatives of T1D patients who are autoantibody positive)^34–37^. After a long duration (>3 years) of T1D, persistent proinsulin expression^38^ and secretion^39^ can be found even without detectable insulin, indicating that some proinsulin-positive β-cells may escape immune attack despite proinsulin processing deficiency^38–42^. Elevated proinsulin is also found in T2D patients at the prediabetic stage in numerous studies^31,43–45^, indicating dysfunctional proinsulin processing at the early phases of β-cell dysfunction^40^. In addition, an increase in the proinsulin level is also associated with an increased risk of cardiovascular disease^46^, insulin resistance^24,47^, and predicts insulin dependency in auto-islet transplant recipients^48^.

We designed a genome-wide CRISPR screen for genes regulating intracellular proinsulin/insulin ratio in a mouse β-cell line (MIN6)^49^. We cross-referenced the hits with the results from an in vivo mouse genetics QTL study for circulating proinsulin levels. We found that the proinsulin regulatory genes and genetic architecture are significantly different from insulin regulatory network and unexpectedly identified *Pdia6* as the strongest hit that positively regulates proinsulin level in both human and mouse β-cells.

## Results

### A genome-wide CRISPR screen for regulators of proinsulin processing

**Fig. 1:**
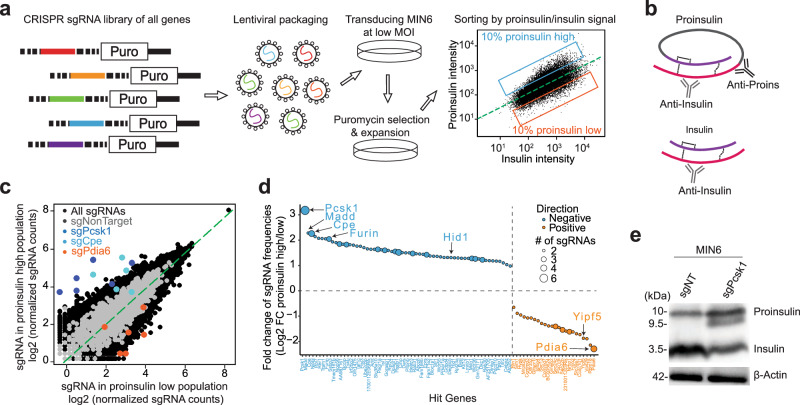
A genome-wide CRISPR screen for proinsulin regulators. **a** The scheme of CRISPR screen. MIN6 cells were transduced with the GeCKOv2 lentiviral library targeting all protein-coding genes at low (Multiplicity of Infection) MOI before puromycin selection. Puromycin-selected cells were stained with both proinsulin and insulin antibodies and sorted into Proinsulin^Hi^ and Proinsulin^Lo^ populations relative to intracellular insulin levels. **b** Specificity of anti-proinsulin and anti-insulin antibodies. **c** Scatter plot comparing the abundance of each gRNA in the Proinsulin^Hi^ and Proinsulin^Lo^ cell populations. Gray dots are non-targeting control sgRNAs. Colored dots show two major negative regulators (Pcsk1 and Cpe) and one positive regulator (Pdia6). **d** 84 candidate proinsulin regulators. The y-axis shows the average fold change of normalized sgRNA frequencies between proinsulin Proinsulin^Hi^ and Proinsulin^Lo^ populations. Circle size indicates the number of supporting sgRNAs. **e** Western blot (in reducing gel, blotted with insulin antibody CST#3014) showing the defects of proinsulin cleavage upon deleting Pcsk1 individually in MIN6 cells. The data were representative of three independent experiments with similar results. Source data are provided as a Source Data file.

We previously published a genome-wide CRISPR screen for “insulin regulators” in mouse MIN6 cells using the genome-scale CRISPR knock-out (GeCKOv2) lentiviral library^50^, which contains 130,209 sgRNAs targeting all the protein-coding genes in the mouse genome (~6 sgRNAs per gene)^51^. The previous insulin screen stained the cells with a pan-insulin antibody (CST, 3014) and then identified small guide RNAs (sgRNAs) that are differentially represented between the Ins^Hi^ (cells with high insulin content) and Ins^Lo^ (cells with low insulin content) populations. However, the previous screen could not reveal the regulators of proinsulin processing because the insulin antibody in our previous screen recognizes both insulin and proinsulin (Fig. 1a).

Here we performed a new screen for regulators of proinsulin processing in the same cell line (Fig. 1a) using a proinsulin antibody (DSHB, GS-9-A8) that specifically recognizes the junction between the B and C chains of proinsulin (Fig. 1b). Since the intracellular insulin and proinsulin levels are highly correlated in the flow cytometry analysis (Fig. 1a), we reasoned that a screen using absolute proinsulin content as the readout will yield the same hits as our previous “insulin regulator” screen (Fig. 1a). To find proinsulin-specific regulators, we stained the cells with both proinsulin and insulin antibodies and defined proinsulin^Hi^ and proinsulin^Lo^ cell populations based on the proinsulin:insulin ratio (Fig. 1b). The screen essentially identifies genes affecting intracellular proinsulin:insulin ratio. For convenience, in this paper, we call these genes “proinsulin regulators” since the readout is the normalized proinsulin content against the total insulin level.

We first compared the normalized read counts from proinsulin^Hi^ and proinsulin^Lo^ cell populations for each sgRNA; some target sgRNAs clearly demonstrated stronger biases than the ~2000 non-target sgRNA controls (Fig. 1c). We then called candidate proinsulin regulators as genes supported by at least two significant sgRNAs (Methods). This resulted in 60 “negative regulators” whose sgRNAs are enriched in the proinsulin^Hi^ population (negative proinsulin regulators reduce proinsulin/insulin ratio), and 24 “positive regulators” whose sgRNAs are enriched in the proinsulin^Lo^ population (positive proinsulin regulators increase proinsulin/insulin ratio) (Fig. 1d and Supplementary Data 1).

Our screen identified known proinsulin regulators. The strongest hits from our screen were *Pcsk1* and *Cpe*, two endoproteases that are responsible for the endoproteolytic cleavage of proinsulin, which are key steps during the conversion to insulin^22,52–56^. Both genes are supported by multiple leading sgRNAs enriched in the proinsulin^Hi^ population (negative regulators, Fig. 1c, d). We verified in MIN6 cells that knocking out *Pcsk1* causes accumulation of proinsulin and a slightly smaller proinsulin intermediate product (Fig. 1e). Interestingly, *Furin* endoprotease is another top negative regulator supported by 3 (out of 5) sgRNAs, although this enzyme is not known to cleave proinsulin (Fig. 1d and Supplementary Data 1). One possibility is that *Furin* may indirectly affect proinsulin processing through an effect on its cellular trafficking^57^. Another top negative regulator, *Madd* has been shown to associate with altered fasting proinsulin level in humans^58^. Additionally, *Yipf5* (positive regulator) and *Hid1* (negative regulator) affect proinsulin trafficking from ER-to-Golgi^59,60^ and the maturation of insulin secretory granules^61^, respectively. Taken together, these results support the ability of our screen strategy to specifically detect defects in proinsulin trafficking.

### Functional annotation of proinsulin regulators

**Fig. 2:**
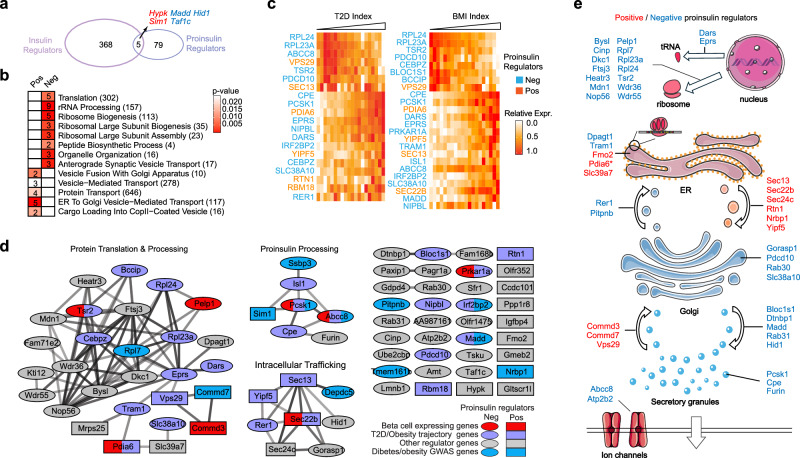
Integrative annotation of proinsulin regulators. **a** Venn diagram showing the overlap between proinsulin regulators and insulin regulators identified from a previous CRISPR screen^51^. The five overlapped genes are shown. Gene names in red show positive proinsulin regulators that increase the proinsulin/insulin ratio, whereas blue indicates negative proinsulin regulators. **b** Gene Ontology enrichment analysis of positive and negative proinsulin regulators using DAVID. Significance was assessed by Fisher’s exact test. Numbers in the heatmap show the screen hits for each category. Numbers in parentheses are all genes belonging to each term. **c** Heatmaps identify proinsulin regulators that are up- or down-regulated in T2D or obesity based on a previously published scRNA-seq trajectory analysis in human islet^51^. **d** STRING analysis of proinsulin regulators. Oval and rectangular shapes indicate negative and positive regulators, respectively. β-cell specific genes (red), T2D and obesity trajectory genes (purple) and diabetes/obesity GWAS genes (blue). **e** Annotation of proinsulin regulators based on their known cellular location or functions in protein synthesis, processing, or transportation.

Only 5 of the 84 proinsulin regulators also are among the 373 insulin regulators identified from a previous orthogonal CRISPR screen^51^ (Fig. 2a). Gene Ontology analysis revealed that translation-related terms are enriched among the negative regulators, such as “ribosome biogenesis” and “ribosomal large subunit assembly” while all other terms are relevant to cellular trafficking (Fig. 2b and Supplementary Data 2). To infer the disease relevance of proinsulin regulators, we compared the hits to the lists of obesity and T2D signature genes identified from our previous single-cell RNA-seq (scRNA-seq) study in human β-cells^51,62^. Twenty-seven of the 84 proinsulin regulators are associated with either T2D or obesity susceptibility genes (Fig. 2c and Supplementary Data 1). Because elevated proinsulin is associated with diabetes^26,27,29–34^, we reasoned that downregulation of negative regulators (such as ribosomal genes *RPL23A*, *RPL24*, and *TSR2*) and upregulation of positive regulators (such as *PDIA6* and *YIPF5*) may exacerbate disease progression by causing an increase in proinsulin. Conversely, upregulated negative regulators (such as *PCSK1* and *CPE*) and downregulated positive regulators (such as *VPS29*) in T2D may alleviate the disease progression (Fig. 2c). We also performed STRING analysis^63^ to group the proinsulin regulators into modules based on regulatory or physical interactions (Fig. 2d) and further highlighted the key information from scRNA-seq analysis for each gene. Consistent with the discussion above, we found three major modules: protein translation, proinsulin processing, and cellular trafficking.

To further elucidate the mechanisms of proinsulin regulation, we grouped the proinsulin regulators based on their cellular locations and reported roles in protein synthesis and trafficking (Fig. 2e). This analysis revealed a highly orchestrated proinsulin regulatory network at different steps of proinsulin processing.

First, as discussed above, endoproteases *Pcsk1*, *Cpe*, and *Furin* are all negative regulators; they reduce the intracellular proinsulin/insulin ratio (Fig. 2e).

Second, the ribosome or tRNA assembly genes that emerged from our screen are all negative regulators of proinsulin (Fig. 2e). The ribosome, especially the large subunit, is not only responsible for protein translation, but also plays an important role in co-translational protein folding^64^. Therefore, we speculate that defects in the translation machinery may lead to the accumulation of immature peptide species recognizable by proinsulin antibody.

Third, the Golgi appears to be the critical organelle controlling the proinsulin/insulin ratio. Trafficking toward the Golgi appears to increase the proinsulin/insulin ratio: screen hits in two branches of trafficking toward the Golgi are all positive regulators (Fig. 2e). The first branch includes genes involved in ER-to-Golgi trafficking, including SNARE protein *Sec22B*^65^, COPII complex proteins *Sec13* and *Sec24C*^66^, reticulon protein *Rtn1*^67^, *Nrbp1*^68^, and *Yipf5*^69^. The second branch includes endosome-to-Golgi retromer complex protein *Vps29* and two of its binding proteins, *Commd3* and *Commd7*^70^. We speculate that these “positive regulators” function to enhance the Golgi as the primary site for proinsulin accumulation; loss of these proteins will decrease the proinsulin/insulin ratio in the cells^71^.

Conversely, trafficking away from the Golgi negatively impacts the proinsulin level in the cells (Fig. 2e). For example, the GO term “anterograde synaptic vesicle transport” represents genes involved in vesicle exocytosis (Fig. 2b). Three hits belonging to this term (*BLOC1S1*, *MADD*, and* DTNBP1*) are all negative proinsulin regulators. *Rab31*^72^ and *Hid1*^61^ are two negative regulators known to regulate the sorting and maturation of secretory exosomes or granules from the Golgi. Defects in these pathways prevent the sorting of proinsulin from the Golgi to the secretory vesicle, where proinsulin is converted to mature insulin. On the other trafficking branch, two negative regulators, *Rer1*^73^ and *Pitpnb*^74^, are both known to be important for Golgi-to-ER retrograde transport (Fig. 2e), which may reduce the accumulation of proinsulin in the Golgi.

Fourth, four other genes localized in the Golgi (*Gorasp1*, *Pdcd10*, *Rab30*, and *Slc38a10*) are negative regulators (Fig. 2e). Notably, *Gorasp1*^75^, *Pdcd10*^76^, and *Rab30*^77^ are involved in maintaining the integrity of the Golgi. Based on the observation that exocytosis reduces cellular levels of proinsulin, we speculate that these genes may also impact insulin secretion. For example, *Gorasp1* also mediates a non-conventional Golgi-independent secretion route^78,79^. Similarly, the K^+^ and Ca^2+^ channel genes *Abcc8* and *Atp2b2* are also negative regulators, and they clearly also play a key role in insulin secretion (Fig. 2e).

Finally, we found genes encoding ER proteins can impact proinsulin both positively and negatively (Fig. 2e). *Dpagt1* is required for protein N-glycosylation and is the target of ER stress-inducing agent Tunicamycin^80^; *Tram1* regulates the translocation of secretory proteins into ER lumen^81^. Both genes are negative regulators and presumably positively regulate insulin secretion. Interestingly, the three positive regulators localized in the ER (*Fmo2*, *Pdia6*, *Slc39a7*) suggest a converged pathway relevant to the formation of protein disulfide bonds: flavin-containing monooxygenase *Fmo2* carries out the thiol oxidation to disulfide, which is necessary for proper protein folding, including insulin; protein disulfide isomerases (PDIs) reconfigure disulfide bonds between cysteine residues. The ER-to-cytosol zinc transporter *Slc39a7* reduces the zinc ion concentration in the ER^82^. Zinc is known to induce conformational changes and inhibit PDIs^83^. Based on the observation that all other positive regulators point to a function involving transport to the Golgi, we speculate that disulfide bond formation may also facilitate proinsulin production and accumulation in the Golgi.

### Integration of CRISPR screen results with an in vivo genetic map of proinsulin QTLs in mice

**Fig. 3:**
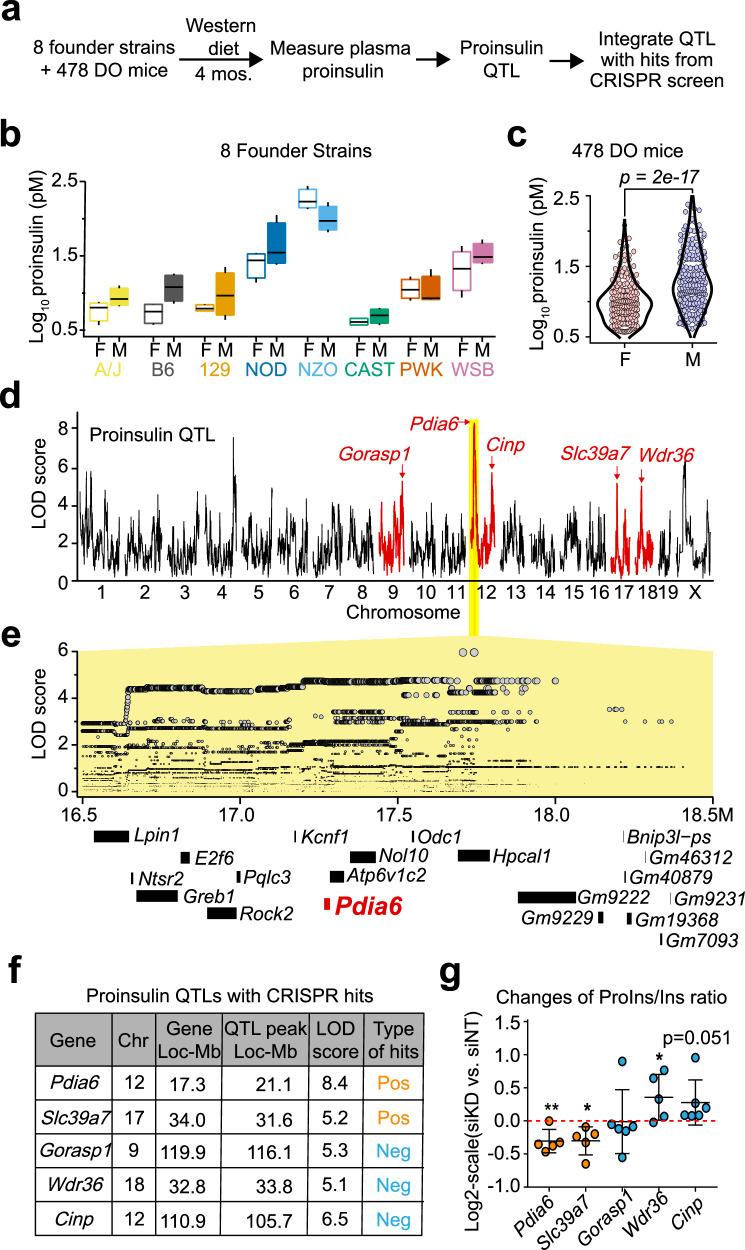
Integrate CRISPR screen results with an in vivo mouse QTL map of circulating proinsulin level. **a** Scheme of mouse QTL study. Eight founder strains and Diversity Outbred (DO) mice were fed with a high-fat, high-sucrose (HF/HS) diet for 16 weeks. Circulating proinsulin level was measured using ELISA and used for QTL analysis in DO mice prior to integration with CRISPR screen hits to nominate gene drivers. **b**,** c** Fasting plasma proinsulin level in both sexes of the eight DO founder strains. A/J (*n* = 8), B6 (*n* = 8), 129 (*n* = 9), NOD (*n* = 8), NZO (*n* = 8), CAST (*n* = 8), PWK (*n* = 9), and WSB (*n* = 8) (**b**) and 478 DO mice (236 female, 242 male). Box plots represent the median (horizontal line), 25th (Q1) and 75th (Q3) percentiles (bounds of the box). Whiskers extended to the observed minima and maxima values within the dataset. Male DO mice have higher proinsulin levels than female DO mice (two-sided Student’s *t*-test). **c** All individual data points are displayed. **d** Genome-wide LOD profile illustrating the proinsulin QTL. Five loci containing hits from the proinsulin CRISPR screen are highlighted in red. **e** SNP association profile for Chr 12 QTL, identifying SNPs most strongly associated with the proinsulin span region containing *Pdia6*. **f** Summary table of five genes nominated from integrative analysis (Methods). **g** Validating CRISPR hits with siRNA. MIN6 cells were transfected with control or target siRNAs, followed by staining for both proinsulin and insulin 72 h later. Median proinsulin/insulin ratio (log2 scale) of all cells from flow cytometry analysis are computed for every experiment. Each data point in the figure represents the change in the proinsulin/insulin ratio between one target siRNA and its parallel control. For each gene, we performed five independent pairs of experiments (siRNA *vs*. siNT control), and the significance for each gene are computed with a one-sided Student’s *t*-test (*p < 0.05, **p < 0.01: paired sample one-tailed *t*-test: siNT vs. siPdia6: *p* = 0.009; siNT vs. siSlc39a7, *p* = 0.017; siNT vs. siGorasp1, *p* = 0.479; siNT vs. siWdr36, *p* = 0.041; siNT vs. siCinp, *p* = 0.051). Data were presented as median ± SD (*n* = 5 independent biological replicates; median fluorescence intensity was calculated from more than 10,000 single cells per replicate). Source data are provided as a Source Data file.

Given the clinical value of monitoring circulating proinsulin to assess in vivo β-cell dysfunction in the context of diabetes^13^, we asked if the proinsulin regulators from the CRISPR screen affect circulating proinsulin levels in vivo. To evaluate this, we exploited genetic diversity within different mouse strains to identify regulators of proinsulin levels, the same approach as we used for circulating insulin^84^ (Fig. 3a and Supplementary Data 3). In this genetic study, we measured circulating proinsulin in both sexes of the eight founder strains of the Diversity Outbred (DO) mouse panel (A/J, B6, 129, NOD, NZO, CAST, PWK, and WSB; *N* ≥ 3/strain/sex for a total of 63 mice), and in 478 DO mice (236 females and 242 males). All mice were metabolically challenged with a high-fat, high-sucrose (HF/HS) diet (western diet) for 16 weeks. Because western diet increases the variability of metabolic phenotypes between individual mice, we and others have routinely used it in many past studies to increase the statistical power of metabolic genetic mappings^84–91^.

Consistent with this idea, under western diet we observed a broad range in circulating proinsulin among the founder strains, yielding ~75-fold difference between CAST (the lowest) and NZO (the highest) mice (Fig. 3b). Interestingly, NZO mice are known to be susceptible to obesity-induced diabetes^92^. This observation is consistent with the reports in humans that increased circulating proinsulin is associated with obesity, impaired glucose tolerance (IGR), and T2D. A two-way ANOVA of the log_2_-transformed values for proinsulin revealed that strain exerts a dominant effect on proinsulin levels (*p* value <10^−15^), while sex (*p* value ~0.03) and sex*strain (*p* value ~0.05) showed a smaller influence. A similarly large range of circulating proinsulin levels was also observed among the DO mice, capturing the full range measured in the founder strains (Fig. 3c). A Student’s *t*-test showed that males have higher proinsulin levels than female DO mice, consistent with our observations in the founder strains. Large strain-dependent differences in phenotypes measured in the founder strains often translate to genetic associations in DO mice. To identify these associations, we performed quantitative trait loci (QTL) analysis on circulating proinsulin levels in DO mice, with sex as an additive covariate (see Methods). We identified two significant QTL (LOD >7) on chromosome Chr 4 (LOD ~7.7) and Chr 12 (LOD ~8.4). Several suggestive QTL (LOD ~6) were also identified, including those on chromosomes 9, 17, and 18, and a second locus on Chr 12. (Fig. 3d).

QTL analysis of physiological traits in DO mice typically yields LOD profiles with Mbp-resolution that may contain multiple genes, depending on the genomic locus^93^. For example, the strongest QTL for proinsulin was on Chr 12 and spanned ~6 Mbp (from ~16–22 Mbp) that contains ~20 protein-coding genes (Supplemental Data 3). We reason that CRISPR screen results will pinpoint the causal genes in the QTL regions, and conversely, the QTL results also support the in vivo function of CRISPR hits. Using this strategy (Methods), we nominated proinsulin regulators for five proinsulin QTL, including three negative regulators (*Wdr36, Gorasp1 and Cinp*) and 2 positive regulators (*Pdia6* and* Slc39a7*) (Fig. 3d–f and Supplementary Fig. 1).

The strongest proinsulin QTL on Chr 12 includes *Pdia6*, which is also the strongest positive regulator from our CRISPR screen (Figs. 1c, d and 3e). Among the five candidates nominated as putative proinsulin regulators, we successfully validated that knocking down three of them (*Pdia6*, *Slc39a7*, and *Wdr36*) significantly changed the proinsulin/insulin ratio in MIN6 cells. Knocking down *Cinp* achieved a slightly less significant *p*-value of 0.051 (Fig. 3g). Notably, *Pdia6-Slc39a7* and *Wdr36-Cinp* are two pairs of genes that may share similar mechanisms during proinsulin processing (Fig. 2e). We therefore concluded that these four genes are bona fide regulators affecting both intracellular and circulating proinsulin levels.

### *Pdia6* dosage impacts proinsulin content and secretion in both human and mouse β-cells

We next focused on validating *Pdia6* because it is the strongest positive proinsulin regulator from the CRISPR screen (Fig. 1d) and the most significant proinsulin QTL (Fig. 3d, e). The identification of *Slc39a7* from both CRISPR and QTL screen further support the role of *Pdia6* because, as discussed above in Fig. 2e, *Slc39a7* is a zinc transporter that regulates protein disulfide isomerases by reducing the zinc concentration in the ER. In fact, *Slc39a7* has been shown to regulate insulin secretion in MIN6 β-cells^94^. *PDIA6* is upregulated in both obesity and T2D trajectories, suggesting a role in disease progression (Fig. 2c).

**Fig. 4:**
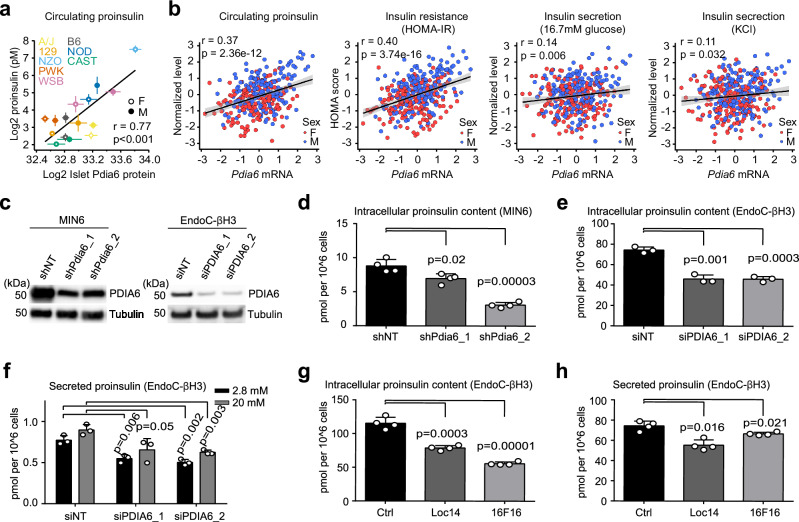
Pdia6 dosage is positively correlated with proinsulin content and secretion. **a** Relationship between islet Pdia6 protein^95^ with circulating proinsulin in both sexes of the eight DO founder strains. Linear regression is used to establish a correlation between Pdia6 and proinsulin. Pearson’s correlation (two-sided) was indicated. Data and error bars are shown as mean ± SEM. **b** Correlation between islet *Pdia6* transcription levels and physiological traits in the DO mice, including plasma proinsulin, HOMA_IR, ex vivo insulin secretion evoked by 16.7 mM glucose, or KCl. Linear regression was performed (gray line, 95% confidence interval shown as gray shading), and Pearson’s correlation (two-sided) was indicated. **c** Western blot showing the efficiency of *PDIA6* knocking down in mouse MIN6 and human EndoC-βH3 cells. **d**,** e** Knocking down *PDIA6* lowers intracellular proinsulin content in both MIN6 and EndoC-βH3 cells, measured by ELISA. Statistical significance was determined using a paired sample *t*-test (two-sided) from four independent biological repeats for MIN6 and three independent biological repeats for EndoC-βH3; Data and error bars are presented as mean ± SEM. **f** Knocking down *PDIA6* decreases the secreted proinsulin in EndoC-βH3 cells. Experiments were performed with three independent biological replicates. Paired sample two-sided Student’s *t*-test was used to compute statistical significance. Data were shown as mean ± SEM. Data were derived from three biological replicates. Student's *t*-test. **g**,** h** PDI inhibitor treatment on EndoC-βH3 cells leads to decreased proinsulin content and secretion. EndoC-βH3 cells were treated with Loc14 (2 μM, 48 h) and 16F16 (1 μM, 48 h). Cells were lysed with RIPA and intracellular proinsulin content was measured by ELISA (**g**). Secreted proinsulin in the culturing media (5.5 mM glucose, 24 h culture) were measured by ELISA (**h**). Data and error bars are presented as mean ± SEM. A two-sided Student’s *t*-test was used to determine statistical significance from four biological repeats. Source data are provided as a Source Data file.

Consistent with the CRISPR screen results that *Pdia6* is a positive proinsulin regulator, the plasma proinsulin levels in the eight founder mice strains are highly correlated with *Pdia6* protein levels in islets^95^; the positive correlation between circulating proinsulin and *Pdia6* transcription is preserved in DO mice (Fig. 4a, b). In contrast, the correlation between *Pdia6* expression and circulating proinsulin is less obvious (Fig. 4b). Interestingly, islet *Pdia6* expression is also positively correlated with insulin resistance (HOMA_IR, *R* = 0.39). Since our CRISPR screen and mechanistic study below indicate a causal role of *Pdia6* in elevating circulating proinsulin level, there are two models to explain the insulin resistance: (i) insulin resistance may upregulate *Pdia6*, which increases the circulating proinsulin; (ii) alternatively, *Pdia6* may increase proinsulin level, which contributes to insulin resistance. It will be interesting to continue investigating the causal relationship between Pdia6 and insulin resistance and T2D.

Knocking down Pdia6 in both mouse (MIN6) and human (EndoC-βH3) β-cell lines (Fig. 4c) significantly decreased the intracellular proinsulin content (Fig. 4d, e and Supplementary Fig. 2a, b), supporting the role of *PDIA6* as a positive proinsulin regulator. We also tested proinsulin secretion with an ELISA assay and found that in both EndoC-βH3 (Fig. 4f) and MIN6 cells (Supplementary Fig. 2c), knocking down *PDIA6* reduces proinsulin secretion, consistent with the finding that *Pdia6* expression is positively correlated with plasma proinsulin level in mice. Finally, two PDI inhibitors (16F16 and Loc14)^96,97^ also lead to decreased proinsulin content and secretion (Fig. 4g, h). These results support a role of *Pdia6* in maintaining proinsulin levels in pancreatic β-cells.

### PDIA6 does not regulate proinsulin trafficking between the ER and the Golgi

**Fig. 5:**
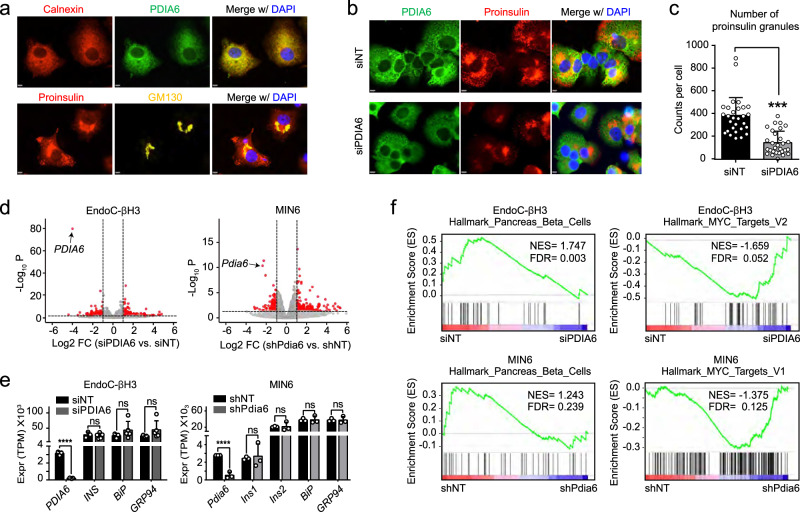
Knockdown of PDIA6 causes limited transcriptomic alterations. **a** Upper panels: Immunofluorescence staining of EndoC-βH3 cells showing the location of *PDIA6* (green) in the ER (marked by *Calnexin* in red). Lower panels: Distribution of proinsulin (red) in Golgi (marked by GM130 in yellow) and secretory granules. Scale bars: 4.4 µm. **b** Staining EndoC-βH3 cells with *PDIA6* and proinsulin antibodies before (siNT) and after (siPDIA6) knocking down PDIA6. Scale bars: 4.4 µm. **c** Quantifications of granule numbers in the EndoC-βH3 cells with (siPDIA6, *n* = 29) or without PDIA6 knockdown (siNT, *n* = 29). Data and error bars are shown as mean ± SD. ***p < 0.001, two-sided Student’s *t*-test. **d** Volcano plots showing differentially expressed genes (DEGs, |log2FC|>1 and *p* < 0.05) from RNA-seq experiments after knocking down *PDIA6* in EndoC-βH3 (left) and MIN6 (right) cells. Significance was determined using a negative binomial model with a Wald test, followed by Benjamini–Hochberg false discovery rate adjustment for multiple comparisons. Adjusted *P* values and log2 (fold change) are displayed. **e** Bar-plots summarize the expression level of *Pdia6* and UPR target genes *Bip* and *GRP94* in EndoC-βH3 (left) and MIN6 cells (right) from RNA-seq data. Data and error bars represent mean ± SD. For EndoC-βH3, *n* = 3 in siNT and *n* = 5 in siPdia6. For MIN6, *n* = 3 for both siNT and siPdia6. Student’s *t*-test (two-sided) was used to compute statistical significance. ****p < 0.0001. **f** GSEA results showing the distribution of genes belonging to each functional term across the profiles of transcriptional changes in EndoC-βH3 (upper panels) and MIN6 (bottom panels) cells after knocking down *PDIA6*. Left panels: Genes in “pancreatic beta-cells” are downregulated in response to PDIA6 knockdown. Right panels: “Myc_target” genes tend to express higher after knocking down *Pdia6*. Source data are provided as a Source Data file.

Immunostaining with *PDIA6* antibody in EndoC-βH3 cells confirmed its localization in the ER (marked by anti-Calnexin, Fig. 5a, upper panels). Staining with proinsulin-specific antibody revealed two main pools of proinsulin: proinsulin co-localizes best with the trans-Golgi Network (TGN) marker GM130 (Fig. 5a, low panels), consistent with our model that the Golgi is the major organelle for proinsulin storage (Fig. 2e); there are also a significant amount of proinsulin present in smaller granules, which presumably are immature secretory granules where proinsulin is further processed into insulin (Fig. 5a, low panels). After knocking down *PDIA6*, although proinsulin is still primarily located in Golgi, the signal strength is attenuated (Fig. 5b), consistent with our model that loss of “positive regulators” reduces proinsulin accumulation in Golgi (Fig. 2e). On the other hand, the number of small proinsulin-containing granules is also significantly lower in *PDIA6*-depleted cells than control (Fig. 5b, c). We speculate that less proinsulin accumulation in the Golgi may relieve the burden of proinsulin processing in the secretory granules, thus decreases proinsulin/insulin ratio. Fewer proinsulin-containing secretory granules also readily explain decreased proinsulin secretion.

We further tested the subcellular distribution of proinsulin by simultaneously labeling proinsulin, ER, and Golgi in EndoC-βH3 cells (Supplementary Fig. 3a). Quantitation analysis confirmed that in wild-type cells, proinsulin-GM130 colocalization is higher than proinsulin-Calnexin colocalization, supporting our conclusion that proinsulin is primarily stored in Golgi (Supplementary Fig. 3b). In PDIA6-depleted cell, although the proinsulin signal appears to be lower, its distribution between ER and Golgi is unaffected according to the quantitative analysis (Supplementary Fig. 3b). In contrast, treatment with BFA (Brefeldin A, an inhibitor of ER-to-Golgi trafficking) leads to significant proinsulin accumulation, and proinsulin-Calnexin colocalization is higher than proinsulin-GM130 colocalization in quantitation analysis (Supplementary Fig. 3b). Taken together, these observations argue against a role of PDIA6 in ER-to-Golgi proinsulin trafficking.

### PDIA6 regulates proinsulin production independent of the UPR pathways

*Pdia6* is a member of the protein disulfide isomerase (PDI) family, which plays a major role in rearranging disulfide bonds that are critical for protein structure and function^98^. In both humans and mice, *PDIA6* point mutations cause diabetes^96,97,99^. The literature suggests two distinct mechanisms by which *Pdia6* may regulate the pool of proinsulin in pancreatic β-cells: (1) A biochemical study showed that *Pdia6* physically interacts with both wild-type proinsulin and mutant Akita proinsulin as a chaperone^100^, suggesting a direct function regulating proinsulin production or processing. (2) An alternative mechanism is that *Pdia6* may repress the unfolded protein response (UPR) pathway by interacting with IRE1α^101^. Specifically, Eletto et al showed that knocking down *Pdia6* in both rat INS-1 cells and mouse MIN6 cells reduces insulin secretion^102^. The authors also reported lower insulin mRNA in rat INS-1 cells upon *Pdia6*-depletion and then attributed this observation to RIDD (regulated IRE-dependent decay) activity. However, the study did not test this conclusion in MIN6 cells^102^.

We performed RNA-seq in both EndoC-βH3 and MIN6 cells before and after knocking down *Pdia6* (Supplementary Data 4). The data confirmed successful knocking down in both cell lines (Fig. 5d), but surprisingly, *PDIA6*-depletion does not significantly affect insulin gene transcription in either cell lines (Fig. 5e), in contrast to its effect in rat INS-1 cells^102^. These results are confirmed by RT-qPCR results (Supplementary Fig. 3c, d). *PDIA6*-depletion also does not increase the transcription of classic UPR target genes (*BiP* and *GRP94*) in either EndoC-βH3 or MIN6 cells (Fig. 5e), suggesting no activation of the UPR pathway. We therefore conclude that *PDIA6*-depletion reduces intracellular proinsulin levels in MIN6 and EndoC-βH3 cells independent of the UPR pathway or RIDD activity.

From the RNA-seq data, we noted ~200 differentially expressed genes in either EndoC-βH3 or MIN6 cells upon *PDIA6*-depletion; GSEA analysis only revealed a limited number of enriched functional terms. “Pancreatic_Beta_Cells” is the term commonly enriched among downregulated genes in both EndoC-βH3 and MIN6 β-cell lines, suggesting compromised β-cell function upon *PDIA6*-depletion (Fig. 5f and Supplementary Data 4). Conversely, “Myc_Targets” is the term commonly enriched among upregulated genes in both cell lines, which is reminiscent of reports that excessive MYC level correlates with decreased levels of β-cell marker genes, inhibition of insulin production, and diabetic conditions^103–108^.

**Fig. 6:**
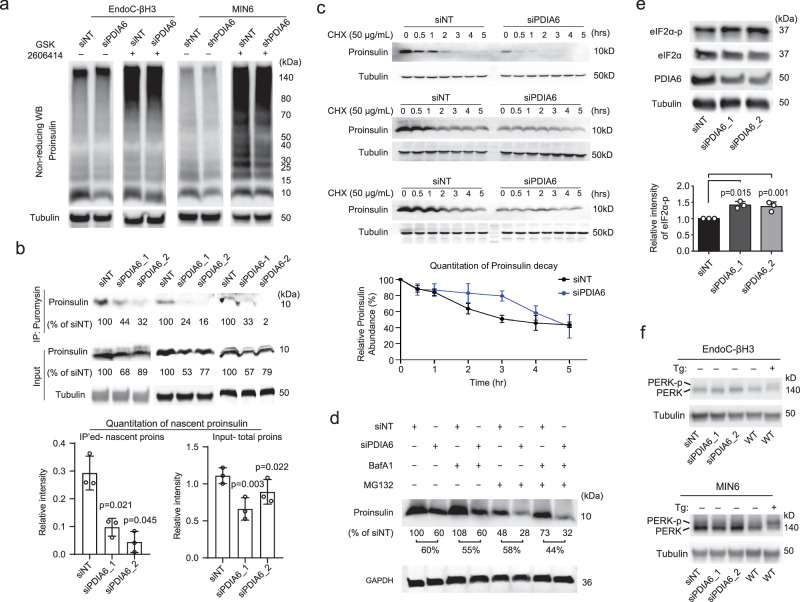
Knocking down PDIA6 impairs proinsulin production instead of degradation, independent of UPR pathways. **a** Western blot for proinsulin in EndoC-βH3 cells (left) or MIN6 cells (right) in non-reducing SDS-PAGE gels before and after treatment with PERK inhibitor (GSK2606414, 2 µM overnight). Bottom bands at 10 kDa are correctly folded monomers. **b** SUnSET assay analysis of proinsulin production. Results from three biological replicates are included. Upper panel: Newly synthesized proteins were immunoprecipitated with anti-puromycin antibody, and Western blots with anti-proinsulin antibody (GS-9-A8) were performed to detect the speed of proinsulin production in EndoC-βH3 cells with or without *PDIA6* knockdown for 5 days. Numbers below the proinsulin band: relative quantitation of band intensity referenced to the first lane after normalization to Tubulin. Lower panels: quantitation of nascent and total (input) proinsulin with data from three independent SUnSET assays. Data and error bars represent mean ± SD. Statistical significance was computed using a two-sided paired *t*-test. **c** Cycloheximide (CHX) chase assay of proinsulin decay. Upper panel: Western blots (three biology replicates) of proinsulin after treating siNT or siPDIA6 EndoC-βH3 cells with CHX (50 µM) for various lengths of time. Lower panel: quantitative curves showing the decay of proinsulin. Proinsulin abundance was normalized to Tubulin, and data points measured the percentage of remaining proinsulin compared to 0-h proinsulin levels. **d** Western blotting showing the proinsulin levels before or after treating with BafA1 (400 nM), MG132 (10 μM), or both overnight. Then percentages of proinsulin were calculated to reflect the effects of *PDIA6*-depletion in each treatment group. **e** Upper panel: Western blot showing an increase of eIF2α Ser51 phosphorylation in EndoC-βH3 cells after knocking down PDIA6. Lower panel: band quantitative from three replicates. Statistical significance was computed using a two-sided Student’s *t*-test. **f** Western blots showed no change of PERK phosphorylation (phosphorylation causes an upward shift of PERK band) after knocking down *PDIA6* in both EndoC-βH3 or MIN6 cells. Tg (Thapsigargin, 400 nM, 1 h) treated cells were used as a positive control. Error bar: mean ± SEM in Student’s *t*-test. Source data are provided as a Source Data file.

We further checked the folding status of proinsulin using non-reducing SDS-PAGE and found no defects in proinsulin folding upon *PDIA6*-depletion or treatment with PDI inhibitors (Fig. 6a and Supplementary Fig. 3e); this is in sharp contrast to the observation when treating the cells with PERK inhibitor (GSK2606414), which leads to accumulation of high molecular weight unfolded proinsulin bearing intermolecular disulfide bonds^109^ (Fig. 6a and Supplementary Fig. 3e). Conversely, we observed an obvious decrease of proinsulin level upon *PDIA6*-depletion or PDI-inhibition, regardless of whether the cells are treated with PERK inhibitor (Fig. 6a and Supplementary Fig. 3e). These results are consistent with the observations from immunostaining experiments (Fig. 5a–c) and suggest that *PDIA6* has a PERK-independent role in regulating proinsulin production. To further test this conclusion, we measured the production rate of proinsulin using the SUnSET system, which labels newly synthesized protein by incorporating puromycin^110,111^. The assay measures the amount of newly synthesized proinsulin protein within 1 h, and we indeed detected significantly reduced proinsulin synthesis after knocking down PDIA6 (Fig. 6b).

Proinsulin undergoes both autophagic and proteasomal degradation in β-cells^112,113^. To test if *PDIA6* affects proinsulin degradation, we performed a cycloheximide (CHX)-chase assay in EndoC-βH3 cells with or without *PDIA6* knockdown. In this assay, we used CHX to block new protein synthesis and then used Western blot to measure the rate of proinsulin decay. We found no evidence of accelerated proinsulin turnover in *PDIA6* knockdown cells (Fig. 6c). We further applied MG132, a proteasome inhibitor, and Bafilomycin A1 (BafA1), an autophagic flux inhibitor, to EndoC-βH3 cells either individually or in combination. BafA1 increases steady-state proinsulin content as previously reported^112^, with or without the presence of MG132 (Fig. 6d). However, MG132 decreases proinsulin content (Fig. 6d), which is also consistent with previous reports that proteasome inhibition leads to lower cellular insulin content and β-cell dysfunction^114–118^. Importantly, *PDIA6*-depletion lowers proinsulin levels regardless of the MG132 or BafA1 treatment (Fig. 6d), suggesting that PDIA6 affects proinsulin levels independent of protein degradation pathways. Taken together, we conclude that the siPDIA6-induced proinsulin reduction is due to decreased protein synthesis, not increased proinsulin degradation.

It is well known that phosphorylation of eIF2α reduces the general rate of protein synthesis in response to the UPR and other stresses^119^. We indeed observed a slight (~1.5-fold) but significant increase in eIF2α (Ser51) phosphorylation after knocking down *PDIA6* (Fig. 6e). It remains to be determined if the eIF2α (Ser51) phosphorylation is adequate to explain the impaired proinsulin production. Importantly, we did not find evidence of PERK activation in *PDIA6*-depleted cells, indicating that the PERK pathway is not responsible for the elevated eIF2α (Ser51) phosphorylation in PDIA6-depleted cells (Fig. 6f). Taken together, we conclude that PDIA6 positively regulates proinsulin production independent of the UPR pathways.

## Discussion

Proinsulin is the pro-hormone of insulin in the pancreatic β-cell. Abnormal proinsulin levels in the blood are an accepted marker of β-cell dysfunction and β-cell stress^35^. Our previous “insulin regulators” screen identified hundreds of genes that regulate total intracellular insulin content^51^, but may have missed the regulators of proinsulin-to-insulin conversion. To fill this gap, here we performed a new “proinsulin regulator” screen using the proinsulin-to-insulin ratio as the readout. Only a few hits are shared between the two screens. Although the proinsulin regulators screen identifies fewer genes, top hits include the well-known proinsulin processing enzymes *Pcsk1* and *Cpe*, indicating that the screen can indeed identify new genes important for β-cell function. Conversely, the “insulin regulators” from our previous screen do not impact the proinsulin-to-insulin ratio, and they are involved in diverse functional categories such as transcription, translation, protein degradation, and mitochondria, etc.^51^. We therefore believe that the “insulin regulator” and “proinsulin regulator” screens are two complementary approaches that represent a complete survey of all targes of interest when considered together. This type of orthogonal screening strategy can be applied to the study of prohormones or peptides in other cell types.

The bottlenecks of proinsulin processing have been extensively investigated. Protein folding and oligomerization, which occur before exit from the ER are widely considered as the rate-limiting steps in the production of secretory proteins^120–123^, including insulin^1,2,124,125^. However, several studies reported accumulation of proinsulin in the Golgi and further suggested that proinsulin exit from the Golgi is also a key regulated step in the insulin biogenesis^11,126–129^. We note that most previous work drew their conclusions through conventional molecular or cell biology approaches, while our unbiased screens provide a novel genomic perspective which clearly highlights the central role of Golgi in the proinsulin processing (Fig. 2e). Remarkably, all hits governing trafficking toward Golgi increase proinsulin/insulin ratio, while hits governing trafficking away from Golgi decrease proinsulin/insulin ratio. This trend can be best explained by a model that the Golgi is the primary organelle for proinsulin storage, which is supported by literature^11,126^ and our own immunostaining data (Fig. 5a and Supplementary Fig. 3a). It is also known that in β-cells, mature insulin is stored in secretory vesicles (SVs). In the ER-Golgi-SV trafficking system, trafficking to the Golgi increases its capacity because lipid bilayer membranes are transported together with cargo proteins; similarly, trafficking away from the Golgi decreases its capacity. We reason that many of the trafficking-related hits alter the capacity of the Golgi, thus governing the proinsulin/insulin ratio in the β-cells. Most of the hits from our screen have not been studied in the context of proinsulin processing.

We compared the proinsulin regulators from the CRISPR screen to the mouse proinsulin QTL results based on the expectation that at least some of the hits may change the plasma proinsulin levels in vivo. In β-cells, because some proinsulin is stored together with insulin in the secretory granules, defects in proinsulin processing may also alter the proinsulin secreted into the bloodstream. It is worth noting that we choose to use “circulating proinsulin” instead of “circulating proinsulin/insulin ratio” for in vivo analysis, as the latter is influenced by multiple factors and might introduce complexity. Specifically, it has been reported that most of the circulating insulin is cleared by hepatocytes at a much faster rate than proinsulin^130–132^, which is the major factor affecting the circulating proinsulin-to-insulin ratio. Therefore, “circulating proinsulin-to-insulin ratio” may be more responsive to liver functions and other factors, while “circulating proinsulin” better reflects β-cell function.

The fact that *Pdia6* is the strongest positive hit from both the CRISPR screen and the in vivo proinsulin-QTL mapping suggests that this gene may directly regulate proinsulin production, folding, or trafficking. *Pdia6* belongs to a family of protein disulfide isomerases (PDIs), which influence disulfide bond formation in the ER. Multiple studies have suggested that PDIs help proinsulin folding and protein exportation from the ER^133–137^. A recent study in a mouse model reported that the deletion of *Pdia1*, a well-studied PDI member in the field of β-cell biology, leads to an increase of proinsulin-to-insulin ratio in β-cells due to the accumulation of unfolded proinsulin^138^. Therefore, in our study, it is unexpected that loss of *Pdia6* decreases the proinsulin-to-insulin ratio and is associated with reduced circulating proinsulin in mice, which is opposite to the assumption that PDIs function through helping proinsulin folding.

Although we did not observe obvious changes of proinsulin localization or folding status in *Pdia6*-depleted β-cells, the most robust and reproducible phenotype was a marked reduction in cellular proinsulin content. We further confirmed a slower proinsulin production in *Pdia6*-depleted cells using the SUnSET assay (Fig. 6b). Interestingly, chemical inhibition of global PDI activity (with 16F16 and LOC14) also reduces proinsulin levels (Fig. 4g, h and Supplementary Fig. 3e), indicating that the PDI activity primarily regulates proinsulin production in β-cells. Notably, *Pdia6* has been reported to repress UPR signaling by interacting with PERK or IRE1α^101^, and consistent with our findings, a previous study also reported lower insulin production and secretion after knocking down *Pdia6* in rat INS-1 cells^102^. The study attributed the reduced insulin level to IRE-dependent mRNA decay (RIDD) of insulin mRNA^102^, but we did not detect PERK phosphorylation or a change of insulin mRNA in either mouse MIN6 or human EndoC-βH3 cells, arguing against the involvement of the UPR pathway as a mechanism in our study. Furthermore, the identification of *Pdia6* as the strongest positive hit from the CRISPR screen supports the idea that it plays a direct role in regulating proinsulin production, rather than acting as an intermediate UPR signaling molecule. One of the possible mechanisms is that the PDI activity may regulate the recruitment of RNA-binding proteins, including the translation machinery, to insulin mRNA^139^. Relevant to this possibility, we observed elevated eIF2α phosphorylation after knocking down *PDIA6*, which may explain the reduced proinsulin translation (Fig. 6e). However, since PERK is not activated, a different kinase may be responsible for the eIF2α phosphorylation in *PDIA6*-depleted cells. Taken together, further studies are still necessary to elucidate the mechanism of how *PDIA6* regulates proinsulin production in pancreatic β-cells.

## Methods

### Cell lines, cell culture conditions

Mouse insulinoma MIN6 cells were cultured in high glucose DMEM with 10% heat-inactivated fetal bovine serum (Gibco), 2 mM GlutMAX (Gibco), 1 mM sodium pyruvate, and 50 μM β-mercaptoethanol. Human EndoC-βH3 were purchased from Human Cell Design and cultured as described before^140^. Briefly, cells were cultured in DMEM containing 5.6 mM glucose, 2% fatty acid-free BSA fraction V, 10 mM nicotinamide, 5.5 mg/ml transferrin, 6.7 ng/ml sodium selenite, Penicillin (100 units/ml)/Streptomycin (100 mg/ml). Cells were passaged and seeded onto matrigel- and fibronectin-coated cell culture dishes every week. About 1 µM tamoxifen (S7827, Selleckchem, Houston, TX, USA) were spiked into culture media to excise transgenes and immortalize cells. For chemical treatments, we treated EndoC-βH3 cells with 16F16 (SML0021, Sigma-Aldrich, St. Louis, MO, USA) at 1 µM for 48 h, with LOC14 (HY-100432, MedChemExpress, Monmouth Junction, NJ, USA) at 2 µM for 48 h, with PERK inhibitor GSK2606414 (S7307, SelleckChem) at 2 µM for overnight, with Bafilomycin A1 (BafA1, S1413, Selleckchem, Houston, TX, USA) at 400 nM for overnight, and/or with MG132 at 10 µM for overnight. 293T cells were cultured in high glucose DMEM with 10% FBS, 2 mM GlutMAX (Gibco, Waltham, MA, USA), and 1 mM sodium pyruvate. All cells were cultured at 37 °C, supplied with 5% CO_2_.

### Mice

Animal care and study protocols were approved by the University of Wisconsin-Madison Animal Care and Use Committee. Mice were housed within the University of Wisconsin-Madison Biochemistry Department vivarium and maintained on a 12-h light/dark cycle (6AM-6PM). Mice were housed under temperature- and humidity-controlled conditions and received ad libitum access to water and food. To generate the Diversity Outbred (DO) founder mice used in this study (at *N* ≥ 3/strain/sex), breeding pairs of the eight founder strains (C57BL/6 J (B6), A/J, 129S1/SvImJ (129), NOD/ShiLtJ (NOD), NZO/HILtJ (NZO), PWK/PhJ (PWK), WSB/EiJ (WSB), and CAST/EiJ (CAST)) were obtained from The Jackson Laboratory (Bar Harbor, ME, USA) and were bred at the University of Wisconsin-Madison Biochemistry Department, except for CAST and NZO, for which all experimental mice were obtained directly from The Jackson Laboratory. Founder mice were group-housed by strain and sex (2–5 mice/cage) except for CAST that required individual housing due to aggressive behavior towards littermates. Details of the DO mouse genetic screen has been previously described^84^. Briefly, the 478 DO mice were obtained from The Jackson Laboratory and, beginning at 4 weeks of age, were maintained on a high-fat/high-sucrose diet (HF/HS) (TD.08811, Envigo Teklad Custom Diet, 44.6% kcal from fat, 14.7% kcal from protein, 40.7% kcal from carbohydrate). Mice were sacrificed at 22 weeks of age, except for NZO males that were sacrificed at 14 weeks, due to high mortality attributable to severe diabetes.

### Plasma proinsulin and other physiological measurements

EDTA plasma was collected retro-orbitally after a 3-h fast at the time of sacrifice to measure proinsulin by ELISA (80-PINMS-E01, ALPCO). For the founder mice, if plasma proinsulin was off the low end of the standard curve for the assay, which was the case for one female CAST, one male CAST, and one female A/J, the value of the lowest standard on the assay was reported (3.69 pM). Plasma proinsulin was off the low end of the curve for 38 of the 478 DO mice. Ex vivo and in vivo insulin secretion and glucose concentration measurement were conducted as reported previously^84^.

### QTL analysis of proinsulin in DO mice and integration with CRISPR screen

Genetic analysis of proinsulin levels essentially followed what we have previously reported for insulin in the same cohort of DO mice^84^. Briefly, circulating levels of proinsulin were transformed by the *rankZ* function in R, yielding a normal distribution. Using R/qtl2^141^, QTL analysis was performed on the rankZ-transformed values of proinsulin, with sex and DO litter as additive covariates.

To nominate potential drivers of proinsulin, we integrated our QTL analysis from the DO mice with the CRISPR screen performed in MIN6 cells (see below). We required a gene to have the following properties at a QTL to be considered for experimental validation: (1) Is genomically located within 4 Mbp of a QTL peak; (2) Expression in islets from DO mice provided in ref. ^88^ is correlated with circulating proinsulin (*r*>|0.3|); and (3) Identified as a proinsulin regulator in the CRISPR screen.

### Pooled sgRNA library amplification

Mouse GeCKOv2 CRISPR sgRNA libraries A and B were purchased from AddGene (AddGene #1000000052) and amplified as described^51^. Briefly, 10 ng of each library was used to transform NEB 10-beta electro-competent cells in 0.1 cm electroporation cuvettes using the MicroPulser (Bio-Rad Laboratories, Hercules, CA, USA) per manufacturer’s instructions. Serious dilution of transfected cells was used to determine transformation efficiency to reach >300X coverage of each clone. After recovery, cells transformed with high efficiencies (>4 × 10^7^ colonies number) were plated into LB petri-dishes (400 µl for each) and cultured overnight. Plasmid extractions were performed using Maxi preparation kits (QIAGEN, Hilden, Germany).

### Lentiviral production and transduction

Around 500 million 293T cells were seeded into fifty 10 cm dishes to reach ~70% confluence the next day. CRISPR library plasmids (4 µg) were co-transfected with 2 µg pVSVG (AddGene 8454) and 2 µg pPAX2 (AddGene 12260) for each plate, using 21 µg polyethylenimine (PEI). Plasmids combo and PEI were each pre-diluted with 500 µl Opti-MEM (Invitrogen, Thermo Fisher Scientific, Waltham, MA, USA), then combined and incubated at room temperature for 10 min. At the same time, 293T cells were changed to 6 ml Opti-MEM before transfection. Ten minutes later, the transfection solution was dropped into each plate. Cells were switched to 10 ml fresh complete medium 6 h later. Supernatant containing virus particles were harvested at 48 and 72 h, and concentrated using ultracentrifugation (25,000×*g*) at 4 °C for 2 h and resuspended with PBS and stored at −80 °C. To calculate virus titer, MIN6 were seeded into 12-well plates (0.1 M per well). Twenty-four hours later, serious dilution of concentrated virus was added into each well for 24 h. Three days later, cells were selected with and without 3 µg/ml puromycin for an additional 3 days, and the cell survival rate were calculated to estimate the viral titer.

### Genome-wide CRISPR screen in MIN6 cells

MIN6 cells (~120 million) were digested and seeded into 10 cm dishes (2.5 M each) the day before transfection. Concentrated GeCKO libraries virus were added into culturing media. The ratio between cells and viruses was estimated with an MOI of 0.3. 24 h after transfection, cells were cultured in fresh complete medium for 3 days and then selected and expanded under 3 µg/ml puromycin for several days. For large-scale screening, 200 million cells were collected and fixed in permeabilization/fixation buffer (BD Biosciences) after expansion, stained and processed for FACS.

### Intracellular staining and FACS

Cells were digested into single cells and fixed with Fixation and Permeabilization buffer (BD Biosciences, #554722) in the dark for 20 min. After that, cells were washed with 1X perm/wash buffer (BD Bioscience, #554723) and stained with insulin antibody (CST, #3014) at 1:100 dilution and proinsulin antibody (DSHB, #GS-9-A8) at 1:100 in 1X perm/wash buffer at 4 °C overnight. The insulin antibody (CST, #3014) recognizes the B-chain of insulin (Fig. 1b) and can detect insulin, proinsulin, and all forms of proinsulin:insulin intermediates. The anti-proinsulin (DSHB, #GS-9-A8) recognizes proinsulin at the B-C junction at AA 26-37 (Fig. 1b) and therefore can detect intact proinsulin and several species of proinsulin intermediates with intact B-C junction (Split-65,66-proinsulin and des-64,65-proinsulin). It does not recognize mature insulin and proinsulin intermediates in which the B-C junction has been cleaved (split-32,33-proinsulin and des-31,32-proinsulin). The specificity of these antibodies has been validated in previous publications^142,143^.

On the second day, cells were washed with 1X perm/wash buffer and stained with AF488-conjugated anti-rabbit IgG secondary antibody (CST, #4412) at 1:200 and APC-conjugated anti-mouse IgG secondary antibody (Invitrogen, #A865) at 1:200 in 1X perm/wash buffer at room temperature in the dark for 30 min. Cells were then washed once with 1X perm/wash buffer and resuspended with PBS. Cells were sorted using BD FACS ARIA (for screening) or simply analyzed by BD LSRII flow cytometer (for individual validation).

### Preparation of genomic DNA for next-generation sequencing

Sorted cells were collected to extract genomic DNA using the Dneasy Blood & Tissue Kit (QIAGEN) per instructions. All sgRNA-expressing cassettes within the genomic DNA are amplified using the mutual GeCKO primers (GeCKO-F: 5’- NNNNNNTCTTGTGGAAAGGACGAAACACCG-3’, GeCKO-R: 5’- TGTGGGCGATGTGCGCTCTG-3’). All harvested gDNA from the screen were amplified by scaling up the reaction number. For each PCR amplification reaction, around 2.5 µg genomic DNA were amplified with Herculase II (Agilent Technologies, Santa Clara, CA, USA) polymerase for an optimized number of cycles (~15–18 cycles). After that, all the PCR products were pooled together and purified with Aline PCR Clean DX magnetic beads. About 50 ng purified PCR products were then ligated to Illumina TruSeq adapters and purified with Aline PCR Clean DX magnetic beads. After that, the library was PCR amplified using TruSeq D&E primers (Illumina, San Diego, CA, USA) for 8–10 cycles. The resulting DNA libraries were then sequenced with standard TruSeq sequencing primers.

### Analysis of pooled CRISPR screen

All raw FASTQ files were analyzed with FASTX-Toolkit (Hannon’s lab). The sgRNA sequences were aligned back to the mouse GeCKO library sequencing with Bowtie2. For each sgRNA, we calculated the *p*-value for its difference in abundance between “relative proinsulin high” and “relative proinsulin low” populations using a binomial test and then the *q*-value was used to compute adjusted FDRs. To call the correspondent genes, we applied the following rules: (1) sgRNAs with FDR <0.01 will be considered significant sgRNAs supporting their enrichment in either population; (2) Genes with more than two supporting sgRNAs in total will be selected as our targets; (3) Any gene with contradictory supporting sgRNAs enriched in both populations will be filtered out. FDRs values of all sgRNAs are listed in the Supplementary Data 1.

### Gene set enrichment and pathway analysis

Gene interaction network were analyzed using the Search Tool for the Retrieval of Interacting Genes/Proteins (STRING) (https://string-db.org/ cgi/input.pl). Interactions with scores greater than 0.40 were applied. Network cosmetics were reconstructed using Cytoscape 3.4.0.

### Gene silencing in MIN6 and EndoC-βH3

SiRNA-based silencing in MIN6 was performed 24 h after seeding into 12-well plates. Generally, siRNAs (150 nM) were pre-incubated with Lipofectamine RNAiMAX (Invitrogen) at room temperature in Opti-MEM reduced serum-free medium for 12 min and then added to cell culture dropwise, and fresh medium changed 24 h later. MIN6 cells were harvested 3 days post-transfection and processed for immunofluorescence staining and flow cytometry. For *PDIA6* knockdown in EndoC-βH3, cells were similarly transfected using Lipofectamine RNAiMAX and harvested 5 days post-transfection. DsiRNAs were purchased from Integrated DNA Technologies (IDT, Coralville, IA, USA) (Pdia6: mm.Ri.Pdia6.13.2, Slc39a7: mm.Ri.Slc39a7.13.1, Gorasp1: mm.Ri.Gorasp1.13.1, Wdr36: mm.Ri.Wdr36.13.2, Cinp: mm.Ri.Cinp.13.2, PDIA6_1: hs.Ri.PDIA6.13.1, PDIA6_2: hs.Ri.PDIA6.13.3, non-targeting control: 51-01-14-04). For shRNA-mediated *Pdia6* knockdown in MIN6, MISSON shRNA bacterial plasmids were purchased from Sigma-Aldrich (shPdia6_1: TRCN0000111770, shPdia6_2: TRCN0000111773) and packaged into lentivirus as described previously. MIN6 cells were infected and selected with puromycin as described before. For sgRNA-mediated gene knockdown in MIN6, LentiCRISPRv2 plasmid were purchased from AddGene (AddGene 52961) and cloned with sgRNA oligos following instructions from AddGene. sgRNA sequence targeting Pcsk1 used in MIN6: Forward: 5’-caccgTACCACTGCTGATTCCACAT-3’, Reverse: 5’-aaacATGTGGAATCAGCAGTGGTAc-3’. sgRNA non-targeting sequencing: Forward: 5’-caccgTGAGAGCAAGGCGCATACGC-3’, Reverse: 5’-aaacGCGTATGCGCCTTGCTCTCAc-3’.

The reason why we do knockdown in MIN6 for 3 days and EndoC-βH3 for 5 days is that MIN6 proliferates faster with a doubling time of approximately 2–3 days. In contrast, EndoC-βH3 is a human pancreatic β-cell line immortalized by an SV40LT^flox/flox^ transgene. This cell line needs to enter a non-proliferative state (by tamoxifen-induced Cre-ERT2-mediated SV40LT-excision) to adopt a β-cell-like state. Based on prior experiences, we chose to do knockdown experiments in EndoC-βH3 cells for 5 days because we expect slower protein turnover rate when cells are in a non-proliferative state.

### RNA isolation, quantitative real-time PCR, RNA-seq and analysis

RNA preparations were performed using the Quick-RNA MicroPrep kit (Zymo Research) per instructions. DNase I was used to remove remaining DNA before reverse transcription with M-MLV reverse transcriptase (Invitrogen) following standard protocol. About 1–2 µg total RNA were used as input for reverse transcription and the cDNA product were diluted with water three to five times. For quantitative QPCR, 1 µl cDNA, 10 µl 2X PerfeCTa SYBR Green SuperMix (Quanta Biosciences, Gaithersburg, MD, USA), 8 µl H_2_O, and 1 µl premixed real-time PCR primers were mixed, and PCR reactions were performed using CFX96 Touch Real-Time PCR detection system (Bio-Rad). Primers used were listed in Supplementary Data 5. For RNA-seq, RNA samples were constructed into a library using the mRNA-seq Lib Prep Kit (RK 20302, ABclonal) per manual instructions, and sequenced using paired-end sequencing. Fastq files were aligned to mouse (mm10) and human (GRCh38) genome reference using HISAT2. Gene expression levels were counted using featureCounts on exonic reads, and DESeq2 was used to perform normalization, principal component analysis and differential gene expression. Analyzed RNA-seq data can be found in Supplementary Data 4. GSEA was performed with MsigDB using the Hallmark Gene Sets option.

### Immunofluorescent (IF) staining

Cells were seeded onto Matrigel-coated cover slides and cultured and processed until the day of the experiment. 4% paraformaldehyde in PBS, pH 7.4, was used to fix cells at room temperature. Cells were washed and incubated with PBS containing 0.25% Triton X-100 for 10 min and washed three times with PBS. Blocking was performed using 1% BSA, 22.52 mg/ml glycine in PBST (PBS + 0.1% Tween 20) for 30 min. Primary antibodies diluted in 1% BSA in PBST were incubated with cells in a humidified chamber at 4 °C overnight. The second day, cells were washed three times in PBS, 5 min each, and incubated with secondary antibodies diluted in 1% BSA in PBST in the dark for 60 min. Cells were washed and incubated with DAPI (1 µg/ml) for 1 min in the dark and washed three times with PBS. Images were performed on a Leica DM6000 upright microscope platform, and FIJI was used for quantification and processing.

For the analysis in Supplementary Fig. 3, EndoC-βH3 cells were incubated with a proinsulin-specific antibody (1:100, GS-9-A8, Developmental Studies Hybridoma Bank, Iowa City, IA, USA), together with antibodies against Calnexin (1:100, 10427-2-AP, Proteintech, Rosemont, IL, USA) and GM130 (1:100, 937002, BioLegend, San Diego, CA, USA). To inhibit ER-to-Golgi trafficking, cells were treated with Brefeldin A (7 µM, HY-16592, MedChemExpress, Monmouth Junction, NJ, USA) for 2 h. Dimethyl sulfoxide (DMSO) was used as the corresponding vehicle control. Proinsulin colocalization with Calnexin or GM130 was quantified to determine the relative colocalized proinsulin volume within the ER and Golgi, respectively. The total intracellular proinsulin volume was normalized to the colocalized proinsulin volume in each compartment. Imaging was performed using the Leica DMi8 Thunder Imager (Leica Microsystems, Wetzlar, Germany), and fluorescence intensity was quantified using ImageJ (National Institutes of Health, Bethesda, MD, USA). Statistical significance was determined using one-way ANOVA with post hoc analysis in GraphPad Prism version 9.2.0 (GraphPad Software, San Diego, CA, USA)

### Proinsulin secretion and content assay in EndoC-βH3

EndoC-βH3 cells were seeded into 12-well plates and changed to fresh medium culture 24 h before. On the day of experiment, cells were starved in low glucose Kreb’s buffer (119 mM NaCl, 5 mM KCl, 1.0 mM CaCl_2_, 1.2 mM KH_2_PO_4_, 1.0 mM MgSO_4_, 24 mM NaHCO_3_, 10 mM HEPES, 0.2% BSA, and 2.8 mM glucose) for 1 h before challenged with either low (2.8 mM) or high (20 mM) glucose fresh Kreb’s buffer for another 1 h. Supernatant was harvested, and cells were washed with PBS and lysed with RIPA for ELISA analysis (Mercodia 10-1118-01). Proinsulin content in MIN6 was analyzed with ELISA (Mercodia 10-1232-01).

### Western blot analysis

Cells were lysed in RIPA (Thermo Scientific) buffer with protease and phosphatase inhibitors on ice for 10 min and further sonicated. Protein lysates were centrifuged for 10 min at 10,000×*g* at 4 °C. Supernatant was collected and quantified by BCA analysis (Thermo Scientific). Samples were prepared in Laemmli sample buffer (Thermo Scientific) and boiled for 5 min at 95 °C. Protein samples were analyzed by SDS-PAGE (4–12% Bis-Tris gel, Thermo Scientific) and transferred to PVDF membranes (Millipore Sigma). The blot was incubated with primary antibodies overnight at 4 °C and on the second day with secondary antibodies for 1 h at room temperature and read by the ChemiDoc Imaging System (Bio-Rad). For non-reducing WB, cells were lysed with RIPA and processed as reducing WB except using a sample buffer without reducing reagent (8% SDS, 20% β-ME, 40% Glycerol, 0.005% Coomassie brilliant blue G-250, 0.25 M Tris-HCl, PH 6.8).

### Surface sensing of translation technique (SUnSET) and Immunoprecipitation

Cells were changed to fresh medium 24 h before. The next day, cells were cultured with fresh medium containing puromycin (10 µg/ml) for 1 h. Cells were washed with ice-cold PBS and lysed with RIPA on ice. Protein lysates were scraped off, sonicated on ice and centrifuged at 10,000×*g* for 10 min at 4 °C. BCA analysis was used to determine the protein concentration. 10% of the protein lysate were collected as the input sample.

The Dynabeads Antibody coupling Kit (Invitrogen, #14311D) were used to conjugate anti-puromycin antibody as per the manufacturer’s instructions. Generally, 1 mg Dynabeads were conjugated with 7.5 µg antibody overnight, rotating at 37 °C and washed multiple times on the second day and stored at 4 °C. On the day of the experiment, 350–400 µg protein lysates were added to the conjugated Dynabeads and rotated at 37 °C for 48 h. Protease inhibitors were added in the middle to inhibit protein degradation during the immunoprecipitation. The incubated beads were then washed three times with the NT2 buffer (50 mM Tris-HCl, pH 7.5, 150 mM NaCl, 1 mM MgCl_2_, and 0.05% NP-40). The precipitates were finally eluted in SDS sample buffer containing sample reducing agent (DTT, Thermo Scientific) at 95 °C for 10 min and analyzed by Western Blot.

### Reporting summary

Further information on research design is available in the Nature Portfolio Reporting Summary linked to this article.

## Supplementary information

Supplementary Information

Description of Additional Supplementary Files

Supplementary Data 1

Supplementary Data 2

Supplementary Data 3

Supplementary Data 4

Supplementary Data 5

Reporting Summary

Transparent Peer Review file

## Source data

Source Data

## Data Availability

Nearly all processed data, including CRISPR screen, mouse genetic screen data, and RNA-seq analysis, are provided in Supplementary Data Tables. Source Data are provided with this paper. The genotypes of the DO mice are previously published^88^ and available at the Dryad drop site: doi:10.5061/dryad.pj105 (data files: Attie Islet eQTL data). The raw RNA-seq and CRISPR screen sequencing data are available in the NCBI GEO database under accession code GSE245846. Source data are provided with this paper.
