# Supplementary material for: Proinsulin regulators identified with CRISPR screen and in vivo mouse QTL mapping
Source: Nat Commun. 2026 Apr 13;17:5159. doi: 10.1038/s41467-026-71726-z (PMC13249969; doi:10.1038/s41467-026-71726-z)

Supplementary Information

Proinsulin regulators identified with CRISPR screen and *in vivo* mouse QTL mapping

Sisi Lai,[1,2,*] Mark P. Keller,[3,*] Jinglin Zhang,[1,*] Zhou Fang,[1,*] Ying Xie,[1,*] Chen Weng,[1,2] Saixian Zhang,[1] Shanshan Zhang,[1,2] Peidong Gao,[1,2] Luxin Ke,[1,2] Yuntong Wang,[1,2] Kelly A. Mitok,[3] Lauren Clark,[3] Kathryn L. Schueler,[3] Hanxiao Liu,[1,2] Betul Hatipoglu,[4,5] Maria Hatzoglou,[1] Yuanyuan Chen,[6] Anath Shalev,[7] Fulai Jin,[1,8,9,#] Alan D. Attie,[3,#] Yan Li[1,#]

1Department of Genetics and Genome Sciences, Case Western Reserve University, Cleveland, Ohio 44122, USA

2The Biomedical Sciences Training Program (BSTP), School of Medicine, Case Western Reserve University, Cleveland, Ohio 44106, USA

3Department of Biochemistry, University of Wisconsin-Madison, Madison, Wisconsin 53706, USA

4Case Western Reserve University School of Medicine, Cleveland, Ohio 44106, USA

5Department of Medicine and Department of Endocrinology, University Hospitals Cleveland Medical Center, Cleveland, Ohio 44106, USA

6Department of Ophthalmology and Department of Pharmacology and Chemical Biology, University of Pittsburgh, Pittsburgh, Pennsylvania 15261, USA

7Comprehensive Diabetes Center, Division of Endocrinology, Diabetes and Metabolism, Department of Medicine, University of Alabama at Birmingham, Birmingham, Alabama 35233, USA

8Department of Computer and Data Sciences and Department of Population and Quantitative Health Sciences, Case Western Reserve University, Cleveland, OH 44106, USA

9Case Comprehensive Cancer Center, Case Western Reserve University, Cleveland, OH 44106, USA

* These authors contribute equally.

# Correspondence authors: Fulai Jin (fxj45@case.edu); Alan D. Attie (attie@biochem.wisc.edu); Yan Li (yxl1379@case.edu).

Supplementary Figures:

Supplementary Figure 1. Proinsulin QTL profiles at four loci which overlap proinsulin regulators from CRISPR screen, related to Figure 3.

Supplementary Figure 2. PDIA6/Pdia6 is a positive proinsulin regulator, related to Figure 4.

Supplementary Figure 3. PDIA6/Pdia6 affects proinsulin production but not its trafficking or folding status, related to Figure 5.

Source Data: Western blot scans for Supplementary Figure 2 and 3.

Supplementary Figure 1

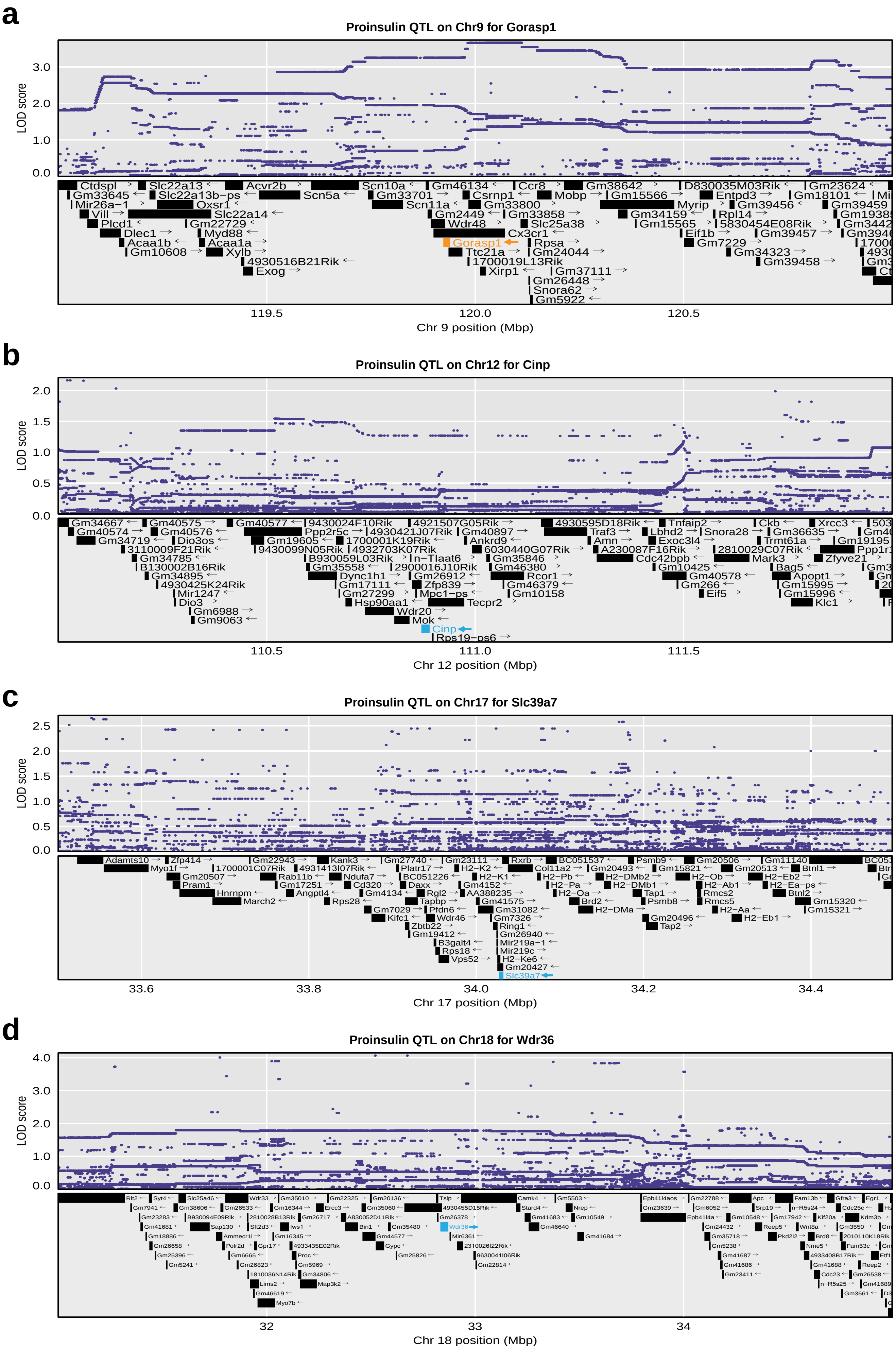

Supplementary Figure 1. Proinsulin QTL profiles at four loci which overlap proinsulin regulators from CRISPR screen, related to Figure 3.

(a-d) The SNPs at Chr 9, 12, 17, and 18 QTLs. Proinsulin regulators in these regions are highlighted.

Supplementary Figure 2

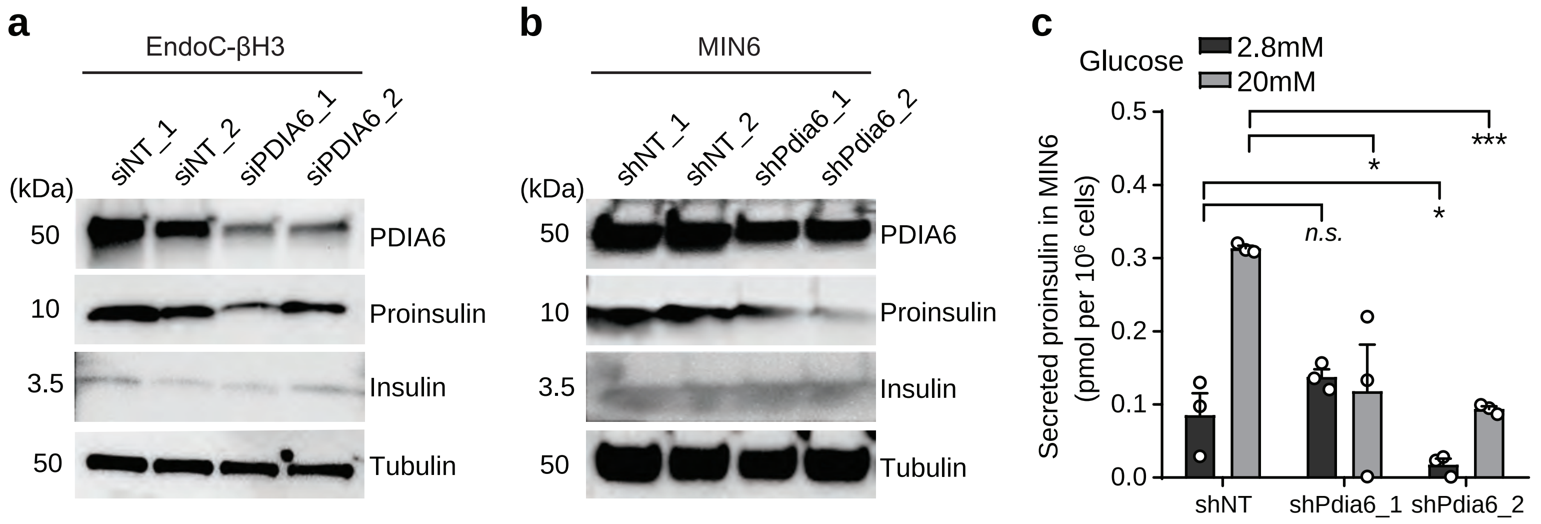

Supplementary Figure 2. *PDIA6*/*Pdia6* is a positive proinsulin regulator, related to Figure 4. (**a-b**) Western blotting showing the protein levels of *PDIA6*/*Pdia6*, proinsulin, and insulin after knocking down *PDIA6*/*Pdia6* in human EndoC-βH3 (**a**) and (**b**) mouse MIN6 cells using siRNAs and shRNAs, respectively. (**c**) Reduced proinsulin secretion by MIN6 cells upon *PDIA6* depletion measured by ELISA. Culture medium was collected three days after shRNA transfection. Each dot represents an individual assay. Error bar: Mean ± S.E.M. siNT or shNT: non-target siRNA or shRNA; *p < 0.05, ***p < 0.001, n.s., not significant in Student's t-test. Source data are provided as a Source Data file.

Supplementary Figure 3

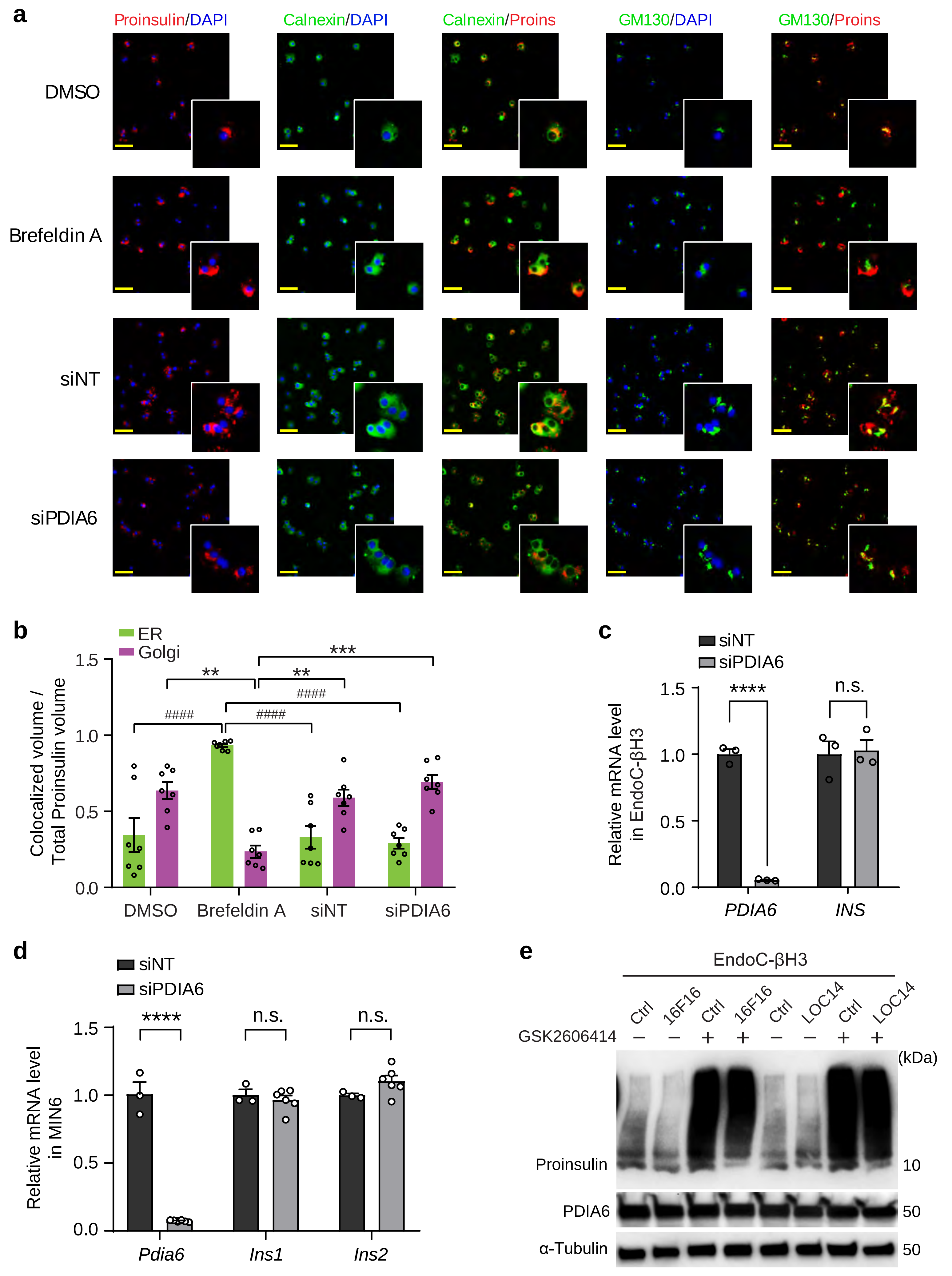

Supplementary Figure 3. *PDIA6*/*Pdia6* affects proinsulin production but not its trafficking or folding status, related to Figure 5.

(**a**) Imaging analysis of proinsulin subcellular location in EndoC-βH3 cells. Cells treated with DMSO, Brefeldin A, or subjected to siRNA transfections are simultaneously stained with anti-Calnexin (ER), anti-GM130 (Golgi), and anti-proinsulin. Error bar, 50 μM. (**b**) Quantitative analyses of proinsulin signal co-localized with Calnexin or GM130. Every dot represents one cell, and the y-axis indicates the fraction of proinsulin signal that co-localized with Calnexin or GM130. Each group has 7 cells for quantification. (**c-d**) RT-qPCR results showing that knocking down *PDIA6*/*Pdia6* do not affect insulin mRNA levels in both EndoC-βH3 (**c**) and MIN6 (**d**) cells. Each dot represents an individual experiment. (**e**) PDI inhibitors (16F16 and LOC14) reduce proinsulin levels in EndoC-βH3 regardless of the PERK inhibition (with GSK2606414). The folding status of proinsulin was tested by Western blot in non-reducing gel, while the levels of PDIA6 and α-Tubulin were measured by Western blot in reducing gel. In all panels, error bar: mean ± S.E.M.; siNT/shNT, siRNA/shRNA with nontarget; ****p < 0.0001, ***p < 0.001, **p < 0.01, ####p < 0.0001, n.s., not significant in Student's t-test. Source data are provided as a Source Data file.

Source Data of WB blot scans

Supplemental Figure 2

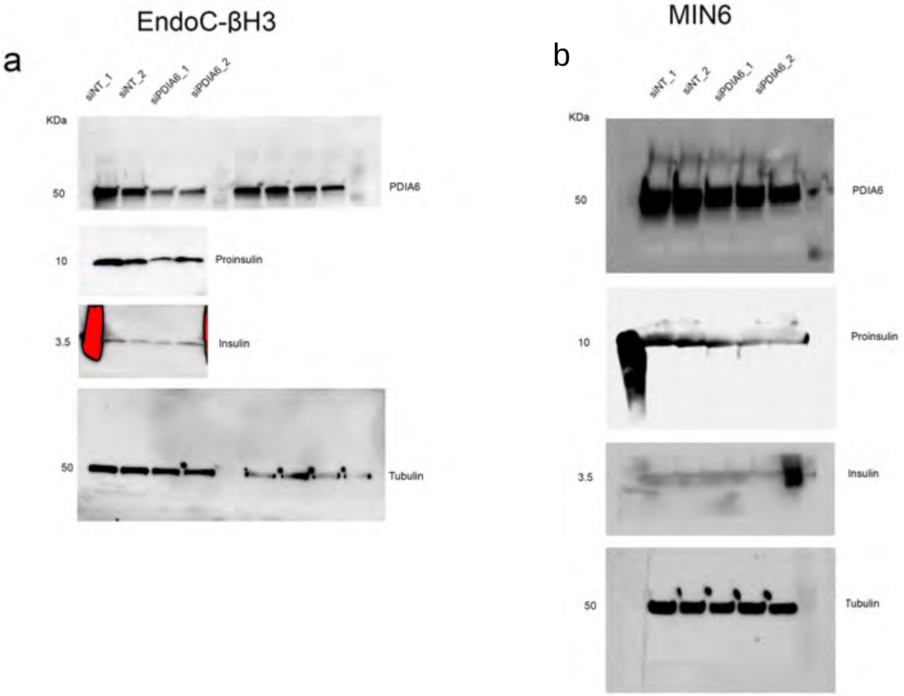

Supplemental Figure 3

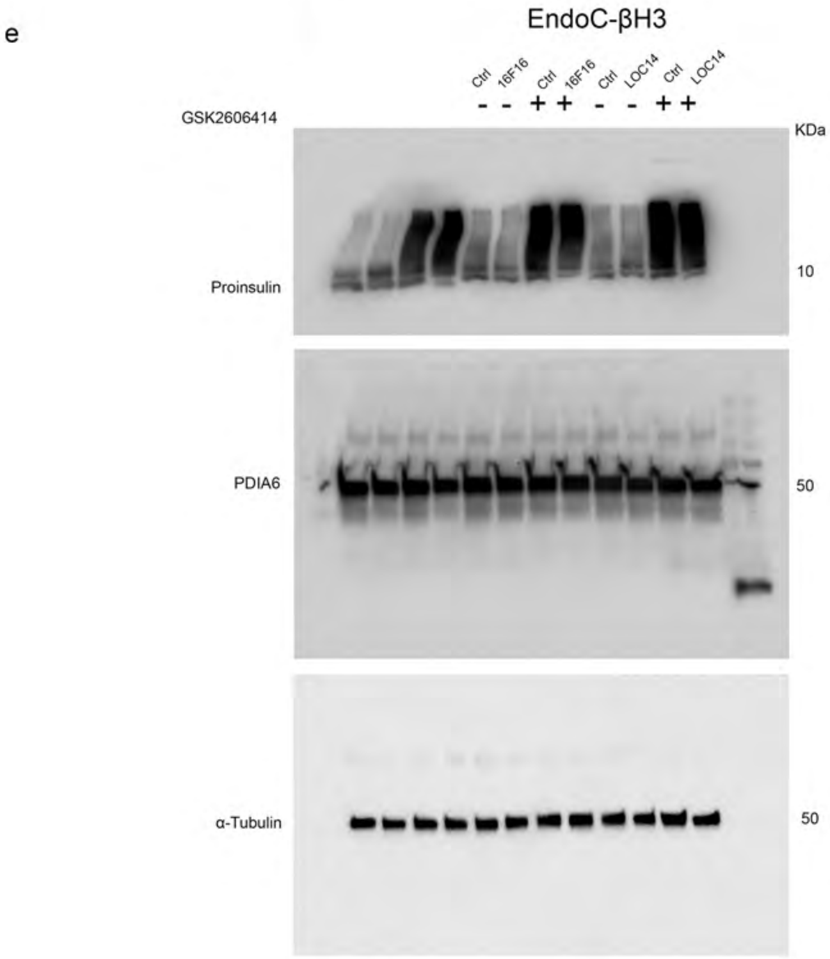

Supplement: Supplementary file 1 — Supplementary Information [file 41467_2026_71726_MOESM1_ESM.pdf]
