# Supplementary material for: Proinsulin regulators identified with CRISPR screen and in vivo mouse QTL mapping
Source: Nat Commun. 2026 Apr 13;17:5159. doi: 10.1038/s41467-026-71726-z (PMC13249969; doi:10.1038/s41467-026-71726-z)
Supplement: Supplementary file 2 — Description of Additional Supplementary Files [file 41467_2026_71726_MOESM2_ESM.pdf]

Description of Additional Supplementary Files

Supplementary Data 1: List of significant genes identified as proinsulin regulators and its overlaps with beta-cell specific genes, disease trajectory genes, GWAS genes and Insulin regulators.

Supplementary Data 2: List of GO terms for identified proinsulin regulators.

Supplementary Data 3: Information about in vivo mice proinsulin QTL study.

Supplementary Data 4: Information about RNA-seq, including normalized reads number, DESeq2 and GSEA results.

Supplementary Data 5: Primers used for real-time PCR of listed genes.
