# Supplementary material for: Proinsulin regulators identified with CRISPR screen and in vivo mouse QTL mapping
Source: Nat Commun. 2026 Apr 13;17:5159. doi: 10.1038/s41467-026-71726-z (PMC13249969; doi:10.1038/s41467-026-71726-z)
Supplement: Supplementary file 8 — Reporting Summary [file 41467_2026_71726_MOESM8_ESM.pdf]

nature portfolio

Corresponding author(s): Fulai Jin, Alan D. Attie, Yan Li

Last updated by author(s): Feb 5, 2026

Reporting Summary

Nature Portfolio wishes to improve the reproducibility of the work that we publish. This form provides structure for consistency and transparency in reporting. For further information on Nature Portfolio policies, see our Editorial Policies and the Editorial Policy Checklist.

Statistics

For all statistical analyses, confirm that the following items are present in the figure legend, table legend, main text, or Methods section.

n/a	Confirmed	
☐	☒	The exact sample size (*n*) for each experimental group/condition, given as a discrete number and unit of measurement
☐	☒	A statement on whether measurements were taken from distinct samples or whether the same sample was measured repeatedly
☐	☒	The statistical test(s) used AND whether they are one- or two-sided<br>*Only common tests should be described solely by name; describe more complex techniques in the Methods section.*
☐	☒	A description of all covariates tested
☐	☒	A description of any assumptions or corrections, such as tests of normality and adjustment for multiple comparisons
☐	☒	A full description of the statistical parameters including central tendency (e.g. means) or other basic estimates (e.g. regression coefficient) AND variation (e.g. standard deviation) or associated estimates of uncertainty (e.g. confidence intervals)
☐	☒	For null hypothesis testing, the test statistic (e.g. *F*, *t*, *r*) with confidence intervals, effect sizes, degrees of freedom and *P* value noted<br>*Give P values as exact values whenever suitable.*
☒	☐	For Bayesian analysis, information on the choice of priors and Markov chain Monte Carlo settings
☐	☒	For hierarchical and complex designs, identification of the appropriate level for tests and full reporting of outcomes
☐	☒	Estimates of effect sizes (e.g. Cohen's *d*, Pearson's *r*), indicating how they were calculated

Our web collection on statistics for biologists contains articles on many of the points above.

Software and code

Policy information about availability of computer code

Data collection	qRT-PCR data were acquired using Bio-Rad (CFX96).<br>Western blot images were acquired using ChemiDoc Imaging System (Bio-rad).<br>Immunofluorescence images were acquired using SP8 (Leica) and DMi8 Thunder imajer (Leica).
Data analysis	R(version4.1.3), GraphPadPrism(version9.2.0), STRING, FASTX-Toolkit (Hannon's lab), Bowtie2, Cytoscape, GSEA, ImageJ

For manuscripts utilizing custom algorithms or software that are central to the research but not yet described in published literature, software must be made available to editors and reviewers. We strongly encourage code deposition in a community repository (e.g. GitHub). See the Nature Portfolio guidelines for submitting code & software for further information.

Data

Policy information about availability of data

All manuscripts must include a data availability statement. This statement should provide the following information, where applicable:

- Accession codes, unique identifiers, or web links for publicly available datasets
- A description of any restrictions on data availability
- For clinical datasets or third party data, please ensure that the statement adheres to our policy

Nearly all processed data, including CRISPR screen, mouse genetic screen data, and RNA-seq analysis, are provided in Supplementary Tables. The genotypes of the

DO mice are previously published and available at Dryad drop site: doi:10.5061/dryad.pj105 (data files: Attie Islet eQTL data). The raw RNA-seq and CRISPR screen sequencing data are available in the NCBI GEO database under accession code GSE245846.

Research involving human participants, their data, or biological material

Policy information about studies with human participants or human data. See also policy information about sex, gender (identity/presentation), and sexual orientation and race, ethnicity and racism.

Reporting on sex and gender	n/a
Reporting on race, ethnicity, or other socially relevant groupings	n/a
Population characteristics	n/a
Recruitment	n/a
Ethics oversight	n/a

Note that full information on the approval of the study protocol must also be provided in the manuscript.

Field-specific reporting

Please select the one below that is the best fit for your research. If you are not sure, read the appropriate sections before making your selection.

☒ Life sciences ☐ Behavioural & social sciences ☐ Ecological, evolutionary & environmental sciences

For a reference copy of the document with all sections, see nature.com/documents/nr-reporting-summary-flat.pdf

Life sciences study design

All studies must disclose on these points even when the disclosure is negative.

Sample size	Sample size was chosen according to previous experience for similar measurements.
Data exclusions	No data were excluded from the analyses.
Replication	Replicates were used in all experiments as indicated in text, figure legends and methods where applicable.
Randomization	All mice and cells were randomized into assay groups.
Blinding	Blinding was not applied to animal study because of grouping information was required.

Reporting for specific materials, systems and methods

We require information from authors about some types of materials, experimental systems and methods used in many studies. Here, indicate whether each material, system or method listed is relevant to your study. If you are not sure if a list item applies to your research, read the appropriate section before selecting a response.

Materials & experimental systems

n/a	Involved in the study	
☐	☒	Antibodies
☐	☒	Eukaryotic cell lines
☒	☐	Palaeontology and archaeology
☐	☒	Animals and other organisms
☒	☐	Clinical data
☒	☐	Dual use research of concern
☒	☐	Plants

Methods

n/a	Involved in the study	
☒	☐	ChIP-seq
☐	☒	Flow cytometry
☒	☐	MRI-based neuroimaging

Antibodies

Antibodies used	Intracellular stain and FACS:<br>Insulin antibody (C27C9), anti-rabbit (Cell Signaling Technology, 3014) at 1:100

	Proinsulin antibody, anti-mouse (DSHB, GS-9-A8) at 1:100<br>Calnexin antibody, anti-rabbit (Proteintech, 10427-2-AP) at 1:100<br>Purified anti-GM130 antibody, anti-rat (BioLegend, 937002) at 1:100<br>Alexa Fluor 488-conjugated anti-rabbit IgG secondary antibody (Cell Signaling Technology, #4412) at 1:200<br>APC-conjugated anti-mouse IgG secondary antibody (Invitrogen, #A865) at 1:200<br>ABflo® 594-conjugated Goat anti-Mouse IgG (H+L) (Abclonal, AS054) at 1:200<br>Alexa Fluor™ Plus 647 goat anti-Rat IgG (H+L) Secondary Antibody (Invitrogen, A48265TR) at 1:200<br><br>Western blotting:<br>Insulin antibody (C27C9), anti-rabbit (Cell Signaling Technology, 3014) at 1:1000<br>Proinsulin antibody, anti-mouse (DSHB, GS-9-A8) at 1:1000<br>PDIA6 antibody, anti-rabbit (Proteintech, 18233-1-AP) at 1:1000<br>Phospho-EIF2S1 (Ser51) Polyclonal antibody (Proteintech, 28740-1-AP) at 1:1000<br>EIF2S1 Polyclonal antibody (Proteintech, 11170-1-AP) at 1:1000<br>PERK antibody (C33E10) anti-rabbit (Cell Signaling Technology, 3192) at 1:1000<br>α-Tubulin Antibody anti-rabbit (Cell Signaling Technology, 2144) at 1:2000<br>mouse anti-rabbit IgG-HRP (Santa Cruz, sc-2357) at 1:2000<br>goat anti-mouse IgG-HRP (Santa Cruz, sc-2005) at 1:2000<br><br>Co-IP:<br>Anti-Puromycin Antibody, (12D10) (Millipore, MABE343) at 1:50
Validation	All antibodies are routinely used in the lab.

Eukaryotic cell lines

Policy information about cell lines and Sex and Gender in Research

Cell line source(s)	Human EndoC-βH3 were purchased from Human Cell Design. Mouse insulinoma MIN6 was a gift from Prof Maria Hatzoglou.
Authentication	None of the cell lines used were authenticated.
Mycoplasma contamination	All cell lines were tested to confirm lack of mycoplasma contamination.
Commonly misidentified lines<br>(See ICLAC register)	None of the cell lines used are commonly misidentified lines.

Animals and other research organisms

Policy information about studies involving animals; ARRIVE guidelines recommended for reporting animal research, and Sex and Gender in Research

Laboratory animals	Diversity Outbred (DO) founder mice was generated in this study (at N≥3/strain/sex). Breeding pairs of the eight founder strains (C57BL/6J (B6), A/J, 129S1/SvImJ (129), NOD/ShiLtJ (NOD), NZO/HILtJ (NZO), PWK/PhJ (PWK), WSB/EiJ (WSB), and CAST/EiJ (CAST)) were obtained from The Jackson Laboratory (Bar Harbor, ME, USA) and were bred at the University of Wisconsin-Madison Biochemistry Department, except for CAST and NZO, for which all experimental mice were obtained directly from The Jackson Laboratory.
Wild animals	n/a
Reporting on sex	In this study, we used both male and female mice.
Field-collected samples	n/a
Ethics oversight	Animal care and study protocols were approved by the University of Wisconsin-Madison Animal Care and Use Committee.

Note that full information on the approval of the study protocol must also be provided in the manuscript.

Plants

Seed stocks	n/a
Novel plant genotypes	n/a
Authentication	n/a

Flow Cytometry

Plots

Confirm that:

- [ ] The axis labels state the marker and fluorochrome used (e.g. CD4-FITC).
- [ ] The axis scales are clearly visible. Include numbers along axes only for bottom left plot of group (a 'group' is an analysis of identical markers).
- [ ] All plots are contour plots with outliers or pseudocolor plots.
- [x] A numerical value for number of cells or percentage (with statistics) is provided.

Methodology

Sample preparation	MIN6 cells were digested into single cells and fixed with Fixation and Permeabilization buffer (#554722, BD bioscience) in the dark for 20 min. Then, cells were washed with 1X perm/wash buffer (BD bioscience) and stained with insulin antibody (CST, #3014) at 1:100 dilution and proinsulin antibody (DSHB, #GS-9-A8) at 1:100 in 1X perm/wash buffer at 4 °C overnight. On the second day, cells were washed with 1X perm/wash buffer and stained with AF488-conjugated anti-rabbit IgG secondary antibody (CST, #4412) at 1:200 and APC-conjugated anti-mouse IgG secondary antibody (Invitrogen, #A865) at 1:200 in 1X perm/wash buffer at room temperature in the dark for 30 mins. Cells were then washed once with 1X perm/wash buffer and resuspend with PBS.
Instrument	BD FACS ARIA for cell sorting, BD LSRII flow cytometer for population analysis
Software	BD FACSDiva
Cell population abundance	*Describe the abundance of the relevant cell populations within post-sort fractions, providing details on the purity of the samples and how it was determined.*
Gating strategy	With FSC and SSC gates, dead cells and doublets/clumps were excluded from the starting cell populations. Isotypic control was used for each sample to define negative and positive populations.

- [x] Tick this box to confirm that a figure exemplifying the gating strategy is provided in the Supplementary Information.
