# Supplementary material for: Proinsulin regulators identified with CRISPR screen and in vivo mouse QTL mapping
Source: Nat Commun. 2026 Apr 13;17:5159. doi: 10.1038/s41467-026-71726-z (PMC13249969; doi:10.1038/s41467-026-71726-z)
Supplement: Supplementary file 9 — Transparent Peer Review file [file 41467_2026_71726_MOESM9_ESM.pdf]

nature portfolio

Peer Review File

Proinsulin regulators identified with CRISPR screen and in vivo mouse QTL mapping

Corresponding Author: Dr Yan Li

This file contains all reviewer reports in order by version, followed by all author rebuttals in order by version.

Version 0:

Reviewer comments:

Reviewer #1

(Remarks to the Author)
The manuscript presented a comprehensive study on the genetic regulation of proinsulin and insulin levels, highlighting several key genes such as Pdia6, Gorasp1, Cinp, Slc39a7, and Wdr36 that significantly influenced these traits. Through detailed QTL mapping across multiple mouse strains, the study identified critical loci on chromosomes 9, 12, 17, and 18 linked to proinsulin levels. By integrating QTL analysis with CRISPR-mediated gene knockdown, authors demonstrated that altering Pdia6 expression significantly affected insulin secretion, underlining its pivotal role in pancreatic beta-cell function. This novel methodological approach not only provided robust functional validation of the genetic associations but also offered potential new targets for diabetes treatment, thereby expanding the current understanding of the molecular mechanisms underlying metabolic disorders.
Major points:
1. In Figure 5a-b, knocking down Pdia6 diminished proinsulin accumulation in the Golgi apparatus and reducing the number of secretory granules. What specific role does Pdia6 play in these processes? Where does the proinsulin that fails to accumulate in the Golgi apparatus go? Is it degraded due to failure in transport to the Golgi apparatus?
2. As shown by the authors, Pdia6 is an ER protein. What are the underlying reasons for the substantial impact of Pdia6 depletion on the accumulation and subsequent secretion of proinsulin in the Golgi and late secretory pathway? Is there a possibility that Pdia6 influences the localization and distribution of proinsulin within the Golgi through involvement in specific signaling pathways, such as calcium signaling or other molecular signaling mechanisms? Could these signaling events potentially modulate the functional status of the Golgi apparatus, thereby affecting the accumulation and processing of proinsulin?
3. In Figure 4g-h, How authors addressed the potential broader effects of PDI inhibitors on the other PDI family members? and how can it be ensured that the observed effects are specifically due to inhibition of Pdia6?
Minor points:
1. Was Fig. 1e run under reducing conditions? If so, the band just below proinsulin is likely the proinsulin intermediate, and band referring as insulin should be the chains of insulin (likely the B chain).
2. The labeled of Pdia6 and tubulin was not consistent in Figure 4c.
3. Western blotting showing both proinsulin and insulin would be helpful in Fig. 4d-e.

Reviewer #2

(Remarks to the Author)

Comments to the authors:
The manuscript investigates the regulation of proinsulin in β cells using mouse and human β cell lines and a genetically diverse mouse model. The authors integrate two powerful whole genome screening approaches, utilizing a genetically diverse mouse model, to identify targets that are involved in the processing of proinsulin within β cells. Results from the screen fit our current models of proinsulin processing, The authors select targets identified in the screen for further validation and demonstrate their effect on β cell proinsulin content and secretion. A particular focus is PDIA6 as the strongest candidate target from the screen and, through mechanistic studies, conclude that PDIA6 likely functions as a direct regulator of proinsulin production.

Major:
1. Please provide additional information about the antibodies used to identify proinsulin and insulin. What is the crossreactivity of the insulin antibody [Insulin (C27C9) Rabbit mAb #3014)] with proinsulin? The proinsulin antibody (DSHB, #GS-9-A8) is reported by the supplier to be specific for proinsulin (https://dshb.biology.uiowa.edu/GS-9A8). Does either react to proinsulin:insulin intermediates?
2. The term "proinsulin regulators" used in the manuscript seems imprecise and problematic. The targets are regulators of the processing and not directly proinsulin. Please discuss the rationale and how this phrasing fits into prior publications. Plus, an extension of this phrasing [line 106, …"negative regulators" enriched in the proinsulinHi population (negative regulators reduce proinsulin level), and 24 "positive regulators" enriched in the proinsulinLo population) (positive regulators increase proinsulin level)] may be confusing to readers.
3. For the CRISPR screen, proinsulin content was normalized to mature insulin content, therefore, hi and lo populations are selected by their proinsulin:insulin ratio. The term 'proinsulin regulators' does not capture this specifically and is interchanged with the 'proinsulin:insulin ratio'. Please discuss.
4. On line 154 begins a discussion on targets that appear to traffic proinsulin towards the Golgi and the resulting effect on the proinsulin:insulin ratio. It is also plausible that reduced trafficking of proinsulin into the Golgi may reduce the amount of mature insulin produced – due to proinsulin availability. With both the proinsulin and mature insulin reservoirs depleted, the proinsulin:insulin ratio could remain unchanged. Please discuss the targets identified by the screen related to this consideration and if the screening approach has overlooked regulators of proinsulin processing.
5. The CRISPR screen used the detection of an altered proinsulin:insulin ratio within β cells, Circulating proinsulin was only used as the metric for the QTL analyses. Please discuss the implications.
6. Please discuss the rationale for subjecting the mouse model to a prolonged metabolic challenge (high sucrose/high fat diet) prior to QTL analyses. Would alterations in proinsulin processing be detectable under normal metabolic conditions? What was the general effect of the metabolic challenge on circulating proinsulin – and how was the effect distributed across the DO cohort?
7. The authors show that PDIA6 expression in islets is positively correlated with both circulating proinsulin and insulin resistance (Fig.4). Given that insulin resistance is linked to T2D pathophysiology and that β cell proinsulin levels are reportedly increased during T2D – this presents insulin resistance as a confounding factor. If PDIA6 is a direct regulator of proinsulin production, one would predict that different levels of PDIA6 alter proinsulin levels independent of the level of insulin resistance. Please discuss.
8. RNA-seq analysis of insulin mRNA expression upon PDIA6 knockdown in the manuscript lies in contrast to that observed in the previously referenced study by Eletto et al., where PDIA6 knockdown decreases insulin mRNA levels at multiple glucose concentrations. Eletto et al quantified insulin mRNA by qPCR. Was the RNA-seq result for insulin mRNA, reported in this manuscript, confirmed by qPCR? Please discuss.
9. The discussion section is very brief. It does not reflect the large amount of data in the manuscript and does not place the work in current and future contexts. These findings are likely applicable to the processing of prohormones or peptides in other cell types.
10. Please discuss the limitations of the screening approach and other items mentioned above.

Minor:
1. In Fig.4, the authors show secreted proinsulin from the EndoC-βH3 cell line, but not from the MIN6 cell line.
2. Fig5 – e) and f) are wrongly labeled in the figure caption

Version 1:

Reviewer comments:

Reviewer #1

(Remarks to the Author)
Thank authors for their efforts. The manuscript was improved. However, two points related my previous major comments need to be addressed:

1. Authors showed a reduction of proinsulin production using an approach of the SuNSET assay in revised Fig. 5h and stated that PDIA6 "DIRECTLY" control proinsulin synthesis. However, how do they exclude the contribution of downregulated proinsulin synthesis due to p-eIF2a shown in revised Fig. 5i ? In addition, it has been shown that PDIs play a role in degradation of proinsulin. To this reviewer, as mentioned in my previous comments, the degradation of proinsulin needs to be measured to determine the contribution of proinsulin degradation to decreased proinsulin content in PDIA6 KD/KO cells. Also, the total proinsulin (input) in Fig 5h was not significantly decreased. Quantification of newly synthesized proinsulin and steady state proinsulin from same experiments are required to validate the findings.

2. In the new revised Fig. S3, only one cell was shown in siPDIA6, in which the volume of GM130 appeared to be reduced. Was it a representative cell? Low magnification images may be helpful to make these clearer.

Reviewer #2

(Remarks to the Author)

The discussion section, though expanded in the revision, is still brief and does not put the new findings in the context of prior work. The discussion section references 7 publications. Three of them (113, 114, 115) refer to insulin clearance work in clinical studies. Two of these refer to prior publications (100,117) about PDIA1. One reference is a clinical study (35). One (51) is a reference to prior publication from this group

Thus, the impact and implications of this work are not clearly stated. How do these findings fit into prior work on proinsulin processing? How does it support or challenge the current model or suggest a new model? For example, on lines 367-375, the manuscript discusses trafficking to and from the Golgi but does not reference prior work or findings or put these findings in the context of what is known about trafficking.

Version 2:

Reviewer comments:

Reviewer #1

(Remarks to the Author)
Thank authors for their efforts. The manuscript was improved. However, two points related my previous comments need to be addressed:

1. The authors performed inhibitor-based experiments to test whether increased degradation contributes to reduced proinsulin levels upon PDIA6 knockdown. While the finding that MG132 and BafA1 treatment did not restore proinsulin levels provides supportive indirect evidence, this approach does not directly assess proinsulin degradation. To support the conclusion that decreased synthesis—rather than enhanced degradation—is responsible, I suggest including direct measurements of proinsulin degradation kinetics, such as pulse-chase or cycloheximide-chase assays.

2. In the revised Fig. 5h, the inclusion of relative band intensities suggests densitometric quantification was performed. However, as it appears to be based on a single experiment without biological replicates or statistical analysis, the conclusions would be strengthened by including multiple data with appropriate quantification. In addition, it would be helpful to clearly state the rationale for selecting the altered time point (5 days post-knockdown instead of 3) in the main text or figure legend, to ensure transparency of the experimental design.

Version 3:

Reviewer comments:

Reviewer #1

(Remarks to the Author)
Authors have addressed my concerns. I have no further questions.

Open Access This Peer Review File is licensed under a Creative Commons Attribution 4.0 International License, which permits use, sharing, adaptation, distribution and reproduction in any medium or format, as long as you give appropriate credit to the original author(s) and the source, provide a link to the Creative Commons license, and indicate if changes were made.
In cases where reviewers are anonymous, credit should be given to 'Anonymous Referee' and the source.
The images or other third party material in this Peer Review File are included in the article's Creative Commons license, unless indicated otherwise in a credit line to the material. If material is not included in the article's Creative Commons license and your intended use is not permitted by statutory regulation or exceeds the permitted use, you will need to obtain permission directly from the copyright holder.
To view a copy of this license, visit https://creativecommons.org/licenses/by/4.0/

We sincerely thank all the reviewers for their time. Their suggestions have significantly improved our manuscript. As detailed below, we have added new experiments (mainly Supp. Fig. 2 and **Supp. Fig. 3**), modified the text to clarify our findings, and significantly expanded the discussion section. We believe we have addressed all reviewer's comments and concerns. In the main text file, we also highlighted major changes in yellow.

Below is the point-by-point response to reviewer's comments.

REVIEWER COMMENTS

Reviewer #1 (Remarks to the Author):

The manuscript presented a comprehensive study on the genetic regulation of proinsulin and insulin levels, highlighting several key genes such as Pdia6, Gorasp1, Cinp, Slc39a7, and Wdr36 that significantly influenced these traits. Through detailed QTL mapping across multiple mouse strains, the study identified critical loci on chromosomes 9, 12, 17, and 18 linked to proinsulin levels. By integrating QTL analysis with CRISPR-mediated gene knockdown, authors demonstrated that altering Pdia6 expression significantly affected insulin secretion, underlining its pivotal role in pancreatic beta-cell function. This novel methodological approach not only provided robust functional validation of the genetic associations but also offered potential new targets for diabetes treatment, thereby expanding the current understanding of the molecular mechanisms underlying metabolic disorders.

We thank the reviewer for the supportive comments about the significance and novelty of our work.

Major points:

1. In Figure 5a-b, knocking down Pdia6 diminished proinsulin accumulation in the Golgi apparatus and reducing the number of secretory granules. What specific role does Pdia6 play in these processes? Where does the proinsulin that fails to accumulate in the Golgi apparatus go? Is it degraded due to failure in transport to the Golgi apparatus?

2. As shown by the authors, Pdia6 is an ER protein. What are the underlying reasons for the substantial impact of Pdia6 depletion on the accumulation and subsequent secretion of proinsulin in the Golgi and late secretory pathway? Is there a possibility that Pdia6 influences the localization and distribution of proinsulin within the Golgi through involvement in specific signaling pathways, such as calcium signaling or other molecular signaling mechanisms? Could these signaling events potentially modulate the functional status of the Golgi apparatus, thereby affecting the accumulation and processing of proinsulin?

Comments #1 and #2 are addressed together here since they are highly related.

We don't think Pdia6 deficiency leads to the accumulation or affects the distribution of proinsulin protein in the cells. Instead, we believe it regulates the translation of proinsulin protein. This is somewhat surprising because the family of protein disulfide isomerases are best known to regulate protein folding. We reach this conclusion due to several reasons:

Firstly, we observed reduced proinsulin signal in Golgi and fewer proinsulin granules upon PDIA6 depletion from immunofluorescence data (**Fig. 5b**), but there is no accumulation of proinsulin in

ER or other organelles. The total amount of proinsulin is effectively reduced (**Fig. 5b**), which is also supported by western blot in **Supplementary Fig. 2a-b** (shown below). These observations better support PDIA6's role in proinsulin translation but do not support its role in proinsulin distribution.

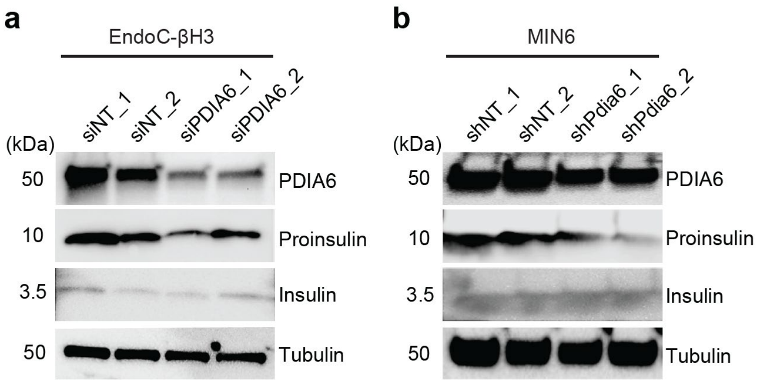

Secondly, we performed the SuNSET assay to test the speed of proinsulin production (**Fig. 5h**). The method measures the amount of newly synthesized proinsulin protein within one hour and consistent with our model of *PDIA6*'s function in translation, we observed reduced proinsulin production in *PDIA6*-depleted cells.

Thirdly, we also added more imaging data to further support our conclusion. In the new **Supplementary Fig. 3a-b** (also shown below), we simultaneously labeled proinsulin, ER (with anti-calnexin), and Golgi (with anti-GM130) to test how PDIA6-depletion or Brefeldin A (BFA, an inhibitor of ER-to-Golgi trafficking) affect the subcellular distribution of proinsulin. (i) In control cells, proinsulin is primarily stored in Golgi, which is consistent with the quantitation analysis showing higher proinsulin-GM130 co-localization than proinsulin-calnexin co-localization. (ii) In PDIA6-depleted cell, although the proinsulin signal appears to be lower, it is still primarily localized in Golgi (**Fig. 5b**) and the distribution between ER and Golgi is not affected according to our quantitative analysis (**Supp Fig. 3b**). (ii) In contrast, BFA treatment causes significant proinsulin

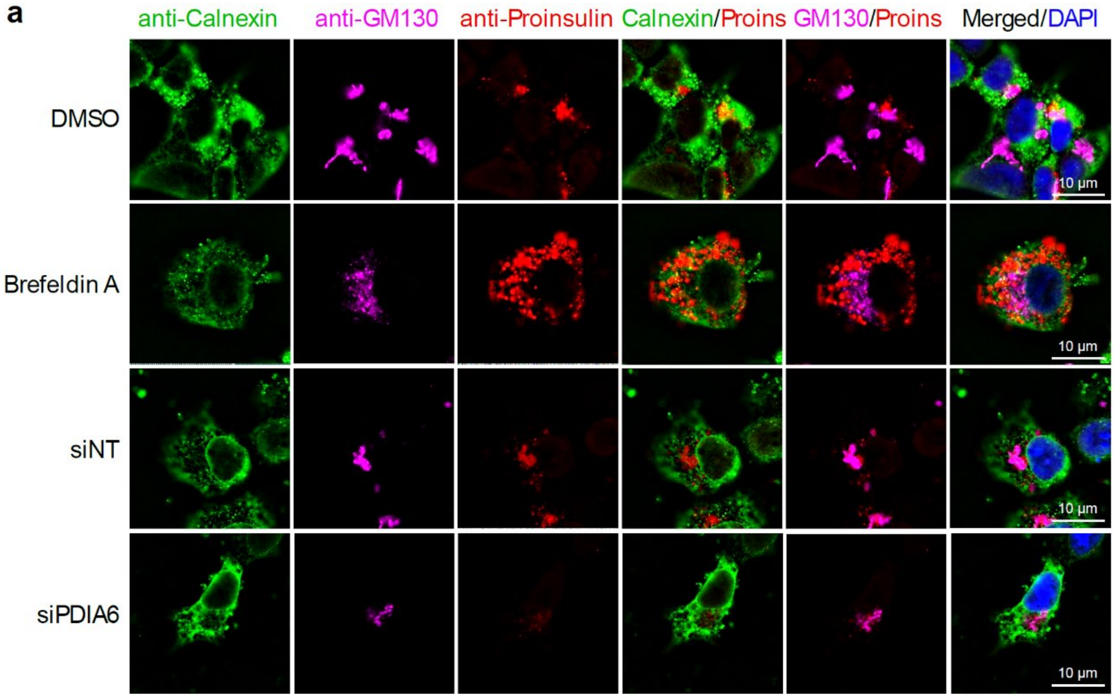

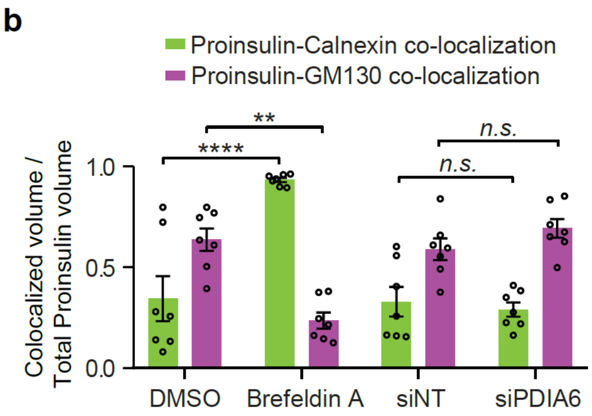

accumulation and in quantitation analysis, proinsulin-Calnexin colocalization is higher than proinsulin-GM130 colocalization, consistent with BFA's function as a blocker of ER-to-Golgi trafficking. All these results argue against a role of PDIA6 in regulating proinsulin trafficking.

We have added the new figures and revised the corresponding text to make our conclusion clearer.

3. In **Figure 4g-h**, How authors addressed the potential broader effects of PDI inhibitors on the other PDI family members? and how can it be ensured that the observed effects are specifically due to inhibition of Pdia6?

This is a great question. Indeed, the protein disulfide isomerase (PDI) inhibitors (Loc14 and 16F16) are expected to inhibit all PDI family members.

We showed in **Fig. 4g-h** that the intracellular and secreted proinsulin levels are lower when treating β-cells with PDI inhibitors, the same phenotypes as we observed in PDIA6-depleted cells. The goal (and conclusion) of this experiment is to show that overall inhibition of PDI activity impacts the proinsulin processing in the same way as depleting PDIA6 in β-cells. To further enhance this conclusion, we now included a new western blot with non-reducing gel (**Supplementary Fig. 3e**, also shown below) showing that PDI inhibitors: (i) do not cause defects in proinsulin folding;(ii) reduce the amount of intracellular proinsulin, regardless of whether PERK activity is inhibited by GSK2606414 (leading to ER stress). Again, this observation is in line with what we observed in PDIA6-depleted cells (**Fig. 5g**). Taken together, although the PDI inhibitors are not specific for PDIA6, our results hint that PDIA6-inhibition is the major reason for the observed drug effects on proinsulin process.

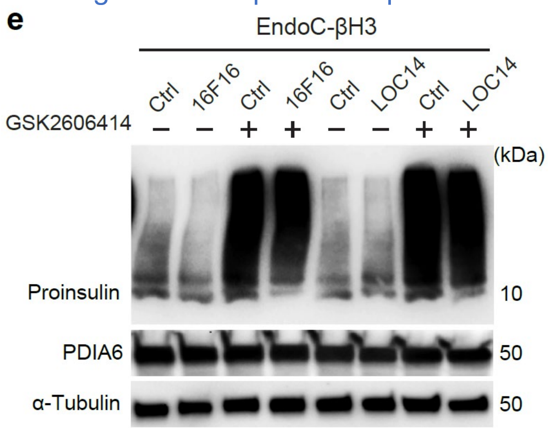

Finally, our findings about PDI inhibitors are important for future applications in basic and potentially translational research, since we show that the inhibitors can conveniently reduce proinsulin content and secretion in the same fashion as PDIA6-depletion.

We also added some additional discussions in the revised ***Discussion*** section (line 401 to 404).

Minor points:

1. Was Fig. 1e run under reducing conditions? If so, the band just below proinsulin is likely the proinsulin intermediate, and band referring as insulin should be the chains of insulin (likely the B chain).

Yes, Western blot in Fig. 1e was indeed run under reducing conditions. The reviewer is correct, the band just below proinsulin is an intermediate at ~9.5 kD, and the band referring as Insulin is the beta chain at ~3.5 kD.

We have added more specific labels ("proinsulin", "intermediates", and "insulin β chain") for the bands in **Fig. 1e**.

2. The labeled of Pdia6 and tubulin was not consistent in Figure 4c.

We thank the reviewer for picking up this issue and have corrected the labeling.

3. Western blotting showing both proinsulin and insulin would be helpful in Fig. 4d-e.

Fig.4d-e measure proinsulin levels using ELISA assay. We have now added Western blots from a replicated experiment in **Supplementary Fig. 2a-b** (shown below). We confirmed lower proinsulin level upon Pdia6 depletion, but the change of insulin band is not obvious.

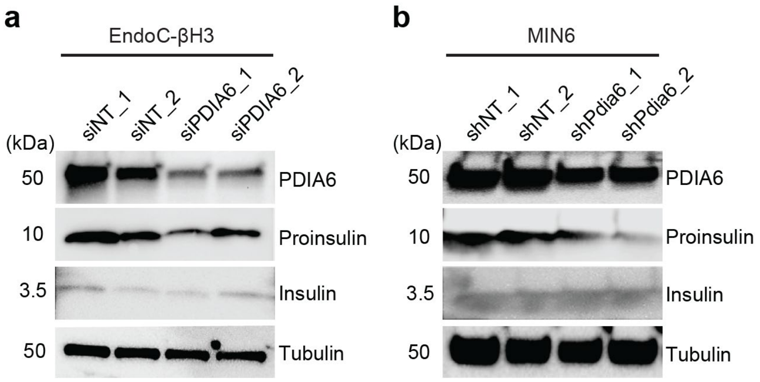

Reviewer #2 (Remarks to the Author):

Comments to the authors:
The manuscript investigates the regulation of proinsulin in β cells using mouse and human β cell lines and a genetically diverse mouse model. The authors integrate two powerful whole genome screening approaches, utilizing a genetically diverse mouse model, to identify targets that are involved in the processing of proinsulin within β cells. Results from the screen fit our current models of proinsulin processing, The authors select targets identified in the screen for further validation and demonstrate their effect on β cell proinsulin content and secretion. A particular focus is PDIA6 as the strongest candidate target from the screen and, through mechanistic studies, conclude that PDIA6 likely functions as a direct regulator of proinsulin production.

Major:

1. Please provide additional information about the antibodies used to identify proinsulin and insulin. What is the crossreactivity of the insulin antibody [Insulin (C27C9) Rabbit mAb #3014)] with proinsulin? The proinsulin antibody (DSHB, #GS-9-A8) is reported by the supplier to be specific for proinsulin (https://dshb.biology.uiowa.edu/GS-9A8). Does either react to proinsulin:insulin intermediates?

Both insulin and proinsulin antibodies are monoclonal antibodies frequently used in β-cell research. We show in **Fig. 1b** that the insulin antibody (CST, #3014) recognizes the B-chain of insulin, which can detect insulin, proinsulin, and all forms of proinsulin:insulin intermediates. In our Western Blot using insulin antibody at reducing condition (**Fig 1e**), we detected a band of B-chain of insulin (3.5 KD) and a band for proinsulin (~10 KD). When knocking down *Pcsk1*, we also detected a band at ~9.5 KD which should represent an intermediate molecule with intact B-C junction.

The anti-proinsulin (DSHB, #GS-9-A8) recognizes proinsulin at the B-C junction at AA 26-37 (**Fig. 1b**) and therefore should recognize intact proinsulin and several species of proinsulin intermediates with intact B-C junction (Split-65,66-proinsulin and des-64,65-proinsulin). It does not recognize mature insulin and proinsulin intermediates in which B-C junction has been cleaved (split-32,33-proinsulin and des-31,32-proinsulin). The specificity of this antibodies has been validated in previous publications (Madsen et al., PMID: 2443408; Asadi et al., PMID: 26216140). In our hands, Western Blot in non-reducing condition(**Fig 5g**) using proinsulin antibody only detect bands at or above ~10 KD, no insulin band (~6.5 KD) were found.

We have added more detail about these antibodies to the ***Methods*** section.

2. The term “proinsulin regulators” used in the manuscript seems imprecise and problematic. The targets are regulators of the processing and not directly proinsulin. Please discuss the rationale and how this phrasing fits into prior publications. Plus, an extension of this phrasing [line 106, …“negative regulators” enriched in the proinsulinHi population (negative regulators reduce proinsulin level), and 24 “positive regulators” enriched in the proinsulinLo population) (positive regulators increase proinsulin level)] may be confusing to readers.

3. For the CRISPR screen, proinsulin content was normalized to mature insulin content, therefore, hi and lo populations are selected by their proinsulin:insulin ratio. The term ‘proinsulin

regulators' does not capture this specifically and is interchanged with the 'proinsulin:insulin ratio'. Please discuss.

Comments #2 and #3 are addressed together here.

We thank the reviewer for raising this question. We have revised the first two paragraphs of the ***Results*** section (line 84 to 101) to further clarify the naming of "proinsulin regulators".

Indeed, as the reviewer pointed out, our screen essentially identifies genes affecting intracellular proinsulin/insulin ratio. The name "proinsulin regulators" is used to avoid long names such as "proinsulin processing regulators" or "regulators of proinsulin:insulin ratio".

On the other hand, since "insulin" is household name that is often used to refer all products from the insulin gene, the name of "proinsulin regulator" hints specificity for proinsulin and its processing to insulin. As we discussed in the paper, we previously published a CRISPR screen for "insulin regulators" (Fang et al., PMID:30865889). In that screen we stained the cells with the pan-insulin antibody (CST, 3014, which recognizes both insulin and proinsulin proteins) and screen for genes that increase or decrease the intracellular insulin levels. However, even if we performed the same screen with a proinsulin-specific antibody, we would have obtained the same hits without "proinsulin specificity" because the intracellular levels of insulin and proinsulin are highly correlated. Proinsulin-specific hits can be only obtained using proinsulin:insulin ratio as the readout. This is why we feel that "proinsulin regulator" is an appropriate name for this study.

Based on the reviewer's suggestions, we also revise the text to clarify that "negative regulators" reduce proinsulin/insulin ratio, and "positive regulators" increase proinsulin/insulin ratio.

4. On line 154 begins a discussion on targets that appear to traffic proinsulin towards the Golgi and the resulting effect on the proinsulin:insulin ratio. It is also plausible that reduced trafficking of proinsulin into the Golgi may reduce the amount of mature insulin produced – due to proinsulin availability. With both the proinsulin and mature insulin reservoirs depleted, the proinsulin:insulin ratio could remain unchanged. Please discuss the targets identified by the screen related to this consideration and if the screening approach has overlooked regulators of proinsulin processing.

Yes, our screen aims to find genes that regulate proinsulin:insulin ratio. If a gene is involved in the proinsulin processing but does not change proinsulin:insulin ratio, it would not show up in our screen.

Theoretically, our proinsulin screen will miss genes that decrease (or increase) the amount of proinsulin and insulin proportionally (the proinsulin:insulin ratio remains unchanged). But those genes can be captured by our previous "insulin regulator" screen (Fang et al., PMID:30865889), which looked for genes that affect the total intracellular insulin level with a pan-insulin antibody. In fact, our previous screen identified many "insulin regulators" that are not "proinsulin regulators". These genes are involved in many different cellular functions such as cell size, transcription, translation, protein degradation, mitochondria, *etc*.

If a gene only affects the trafficking to Golgi but does not affect total intracellular insulin amount, we shall expect the accumulation of proinsulin elsewhere such as in ER. For example, we observed the accumulation of proinsulin in ER after treating cells with Brefeldin A, an ER-to-

Golgi inhibitor (the first two rows of **Supplementary Fig. 3a**, also shown below). In this scenario, the proinsulin:insulin ratio will still increase.

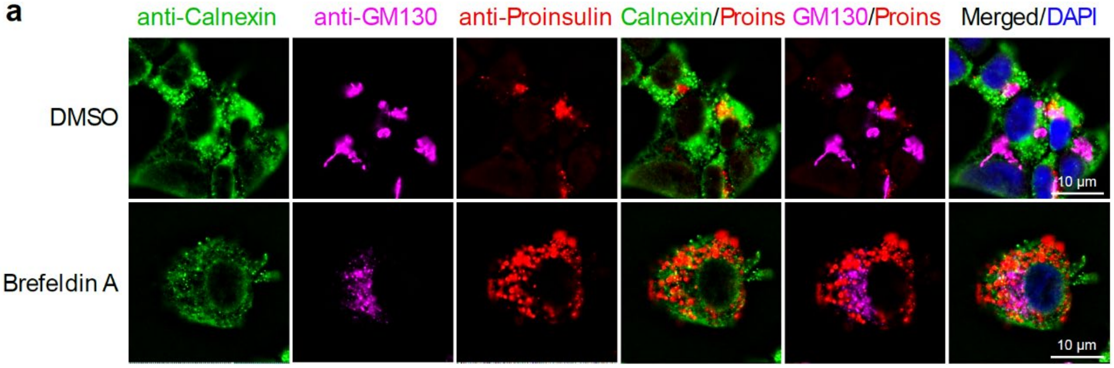

We therefore believe that the “insulin regulator” and “proinsulin regulator” screens are two complementary approaches that represent a complete survey of all targes of interest when considered together.

In the first paragraph of the ***Discussion*** section (line 353 to 365), we have added more discussions about how the two screen are complementary to each other.

5. The CRISPR screen used the detection of an altered proinsulin:insulin ratio within β cells, Circulating proinsulin was only used as the metric for the QTL analyses. Please discuss the implications.

In short, we choose to perform QTL analysis of circulating proinsulin level because it is a good measurement of the β-cell function in proinsulin processing, perhaps better than the proinsulin:insulin ratio in the blood.

In β-cells, a small fraction of unconverted proinsulin is stored together with insulin in the secretory granules. Upon glucose stimulation, proinsulin will be co-secreted into the bloodstream. Defects in proinsulin processing will increase the fraction of proinsulin in the secretory granule and elevate the circulating proinsulin level.

However, the control of proinsulin/insulin ratio in blood stream is more complex. Specifically, the clearance of insulin is much faster than proinsulin in the body, which is the major factor affecting the circulating proinsulin/insulin ratio. It has been estimated that 50% of circulating insulin is cleared by hepatocytes in liver (PMID:30968756). The rest insulin hormone is eventually disposed in kidney at a much slower rate. In contrast, the primary clearance site of proinsulin is probably kidney because the hepatic clearance of proinsulin is much slower than insulin (PMID:1516487,10837704). Due to these reasons, circulating proinsulin/insulin ratio is more susceptible to liver and kidney functions, while circulating proinsulin level may better reflect β-cell function.

We have added a few sentences in the ***Discussion*** section (line 380 to 385) discussing about using circulating proinsulin instead of proinsulin:insulin ratio for QTL analysis.

6. Please discuss the rationale for subjecting the mouse model to a prolonged metabolic challenge (high sucrose/high fat diet) prior to QTL analyses. Would alterations in proinsulin processing be detectable under normal metabolic conditions? What was the general effect of the metabolic challenge on circulating proinsulin – and how was the effect distributed across the DO cohort?

The DO cohort is generated from 8 inbred founder mouse strains. The basis of the genetic mapping with DO mice is that the founder strains show different phenotypes under the same condition. Western diet is commonly used to increase the statistical power of metabolic genetic mappings because it increases the variability of metabolic phenotypes between individual mice. For example, western diet was used to challenge >100 inbred mouse strains to genetically map a spectrum of metabolic phenotypes including body composition, obesity, glucose homeostasis, *etc.* (PMID: 23312289, 17317875, 26381349, 32958938). We (Attie lab) have also published multiple genetic studies using DO mice, including islet eQTL, insulin secretion, gut microbiota composition and bile acid profile, liver and plasma lipid profiles (PMID: 29567659, 31343992, 31465442, 25001233), all under western diet. Consistent with this idea, under western diet we observed a large range in circulating proinsulin among the founder strains (**Figure 3b**); a similarly large range of circulating proinsulin levels was also observed among the DO mice (**Figure 3c**).

Regarding the question if we can identify the same proinsulin-QTL loci under normal diet. Unfortunately, we cannot directly answer this question without regenerating the entire DO mice cohort under normal diet and analyzing their metabolic features, which is prohibitively difficult. However, it is plausible that under normal diet, the variability of circulating proinsulin will be much smaller. Due to the smaller effect size, larger sample size (more mice) will be necessary to map statistically significant associations.

Along the same line of discussion, we expect that western diet will impact circulating proinsulin level at different amplitude depending on the strains, which requires more extensive study. In **Fig. 3b**, we report that under western diet, blood proinsulin is highest in NZO mice, regardless of sex. Interestingly, NZO mice are known to be susceptible to obesity-induced diabetes. This observation is consistent with the reports in human that increased circulating proinsulin is associated with obesity, impaired glucose tolerance (IGR), and T2D.

We have added a few sentences in the ***Result*** section (line 204 to 212) about western diet.

7. The authors show that PDIA6 expression in islets is positively correlated with both circulating proinsulin and insulin resistance (Fig.4). Given that insulin resistance is linked to T2D pathophysiology and that β cell proinsulin levels are reportedly increased during T2D – this presents insulin resistance as a confounding factor. If PDIA6 is a direct regulator of proinsulin production, one would predict that different levels of PDIA6 alter proinsulin levels independent of the level of insulin resistance. Please discuss.

We thank the reviewer for an inspiring question. Indeed, there is an inter-correlation between Pdia6 expression, insulin resistance, and circulating proinsulin. The reviewer raised a hypothesis that insulin resistance may cause elevated proinsulin and Pdia6 upregulation at the same time, which explains the correlation between Pdia6 and proinsulin levels.

If this hypothesis is true, many (if not all) insulin-resistance genes should regulate circulating proinsulin and become proinsulin QTLs. However, we do not recognize many insulin resistance

genes or T2D risk genes from the top proinsulin QTLs. Furthermore, if Pdia6 became a proinsulin QTL through an indirect mechanism (insulin resistance), it is unlikely to be the strongest proinsulin QTL.

Our CRISPR screen and mechanistic experiments in cell lines indicate a causal role of *Pdia6* in elevating circulating proinsulin level. There are two models to explain the insulin resistance: (i) Insulin resistance may upregulate Pdia6, which increases the circulating proinsulin; (ii) alternatively, Pdia6 may increase proinsulin level, which contributes to insulin resistance. In both models, we should still observe the inter-correlation between Pdia6 expression, insulin resistance, and circulating proinsulin. It will be interesting to continue investigating the causal relationship between Pdia6 and insulin resistance/T2D.

We have added some discussion into the ***Result*** section about how to interpret the results about insulin resistance (line 254 to 260).

8. RNA-seq analysis of insulin mRNA expression upon PDIA6 knockdown in the manuscript lies in contrast to that observed in the previously referenced study by Eletto et al., where PDIA6 knockdown decreases insulin mRNA levels at multiple glucose concentrations. Eletto et al quantified insulin mRNA by qPCR. Was the RNA-seq result for insulin mRNA, reported in this manuscript, confirmed by qPCR? Please discuss.

We thank the reviewer for the question. Indeed, we noticed the discrepancy between our data and Eletto *et al.*

Eletto et al. observed that after knocking down *Pdia6*, insulin secretion becomes lower in both rat INS-1 cells and mouse MIN-6 cells. The authors further reported lower insulin mRNA in rat INS-1 cell upon *Pdia6*-depletion and then attributed this observation to RIDD (regulated IRE-dependent decay) activity. However, they did not test this conclusion in MIN-6 cells.

We did not observe a decrease of insulin mRNA in either MIN-6 or human EndoC-βH3 cells from the RNA-seq data, even though the *PDIA6*-depletion is clearly successful (**Fig. 5d,e**). We now also added RT-qPCR data in **Supplementary Fig. 3c-d** (also shown below) and confirmed the RNA-seq results. Based on these results, we conclude that at least in MIN-6 and EndoC-βH3 cells, *PDIA6*-depletion reduces intracellular proinsulin levels through other mechanisms.

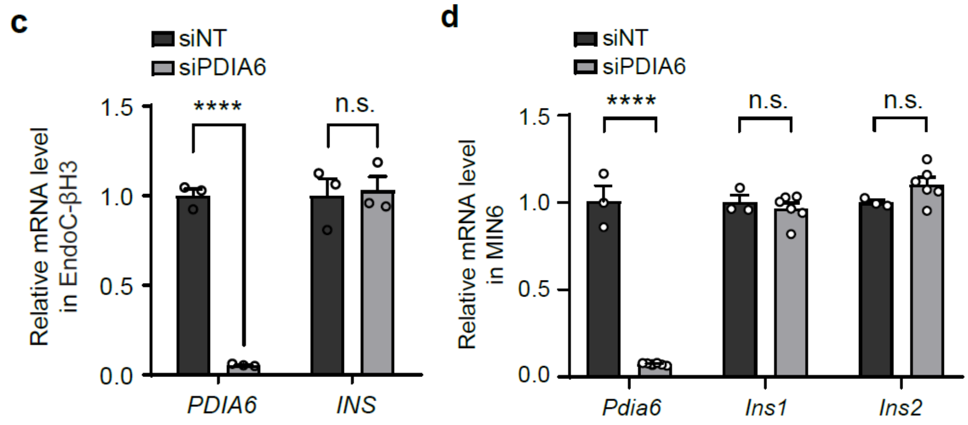

We added more discussion about these discrepancies in the ***Result*** section (line 305 to 314).

9. The discussion section is very brief. It does not reflect the large amount of data in the manuscript and does not place the work in current and future contexts. These findings are likely applicable to the processing of prohormones or peptides in other cell types.

We thank the reviewer for the suggestions and have now significantly expanded the ***Discussion*** section.

10. Please discuss the limitations of the screening approach and other items mentioned above.

We thank the reviewer for the suggestions and have added more discussions in both ***Results*** and ***Discussion*** sections to address all the comments above.

Minor:
1. In Fig.4, the authors show secreted proinsulin from the EndoC-βH3 cell line, but not from the MIN6 cell line.

We have added a figure panel (**Supplementary Fig. 2c**, shown below) showing that secreted proinsulin also decreases in MIN6 cells.

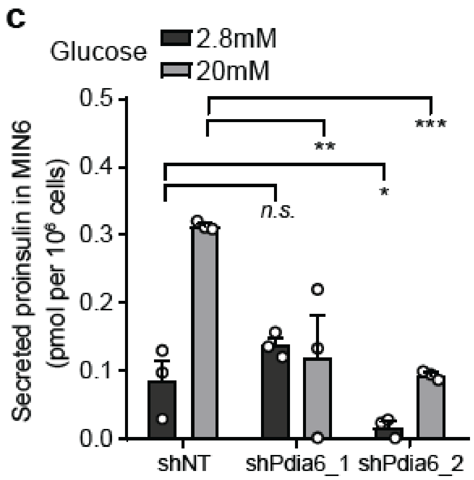

2. Fig5 – e) and f) are wrongly labeled in the figure caption

We thank the reviewer for picking up this error and have corrected the figure caption.

We sincerely thank the reviewers for their time and comments and have addressed the remaining concerns below. In the following, the reviewers' comments are in blue, and our responses are in black.

Reviewer #1 (Remarks to the Author):

Thank authors for their efforts. The manuscript was improved. However, two points related my previous major comments need to be addressed:

1. Authors showed a reduction of proinsulin production using an approach of the SuNSET assay in revised Fig. 5h and stated that PDIA6 "DIRECTLY" control proinsulin synthesis. However, how do they exclude the contribution of downregulated proinsulin synthesis due to p-eIF2a shown in revised Fig. 5i ?

In the previous version we wrote:

"…We indeed observed a slight (~1.5 fold) but significant increase of eIF2α (S51) phosphorylation after knocking down PDIA6 (Figure 5i). However, it remains to be determined if this observation is adequate to explain the impaired proinsulin production. Furthermore, we did not find evidence of PERK activation in PDIA6 depleted cells, indicating that PERK pathway is not responsible for the elevated eIF2α (S51) phosphorylation in PDIA6-depleted cells (Figure 5j). Taken together, we conclude that PDIA6 positively regulates proinsulin production independent of the UPR pathways; instead, the role of Pdia6 as a proinsulin chaperone may explain its function in β-cells..."

We apologize that the highlighted sentence in the context of this paragraph is misleading and not what we want to claim. We do not intent to rule out the contribution of p-eIF2α although we cautioned more work is necessary to determine if the increased p-eIF2α is adequate to explain the reduced proinsulin synthesis. We have now deleted the highlighted sentence and slightly modified the rest of this paragraph as the following:

"…We indeed observed a slight (~1.5 fold) but significant increase of eIF2α (S51) phosphorylation after knocking down PDIA6 (Figure 5j). It remains to be determined if the eIF2α (S51) phosphorylation is adequate to explain the impaired proinsulin production. Importantly, we did not find evidence of PERK activation in PDIA6 depleted cells, indicating that PERK pathway is not responsible for the elevated eIF2α (S51) phosphorylation in PDIA6-depleted cells (Figure 5k). Taken together, we conclude that PDIA6 positively regulates proinsulin production independent of the UPR pathways…"

We also revised the following sentences in the discussion session to further clarify:

*"…the fact that Pdia6 is the strongest positive hit from the CRISPR screen also suggests that this gene may play a direct role regulating proinsulin production, rather than acting as an intermediate UPR signaling molecule. One possible mechanism is that the PDI activity may regulate the recruitment of RNA binding proteins, including the translation machinery, to insulin mRNA[139]. Relevant to this possibility, we observed elevated eIF2α phosphorylation after knocking down PDIA6, which may explain the reduced proinsulin translation (**Fig. 5j**). However, since PERK is not activated, a different kinase may be responsible for the eIF2α phosphorylation in PDIA6-depleted cells…"*

In addition, it has been shown that PDIs play a role in degradation of proinsulin. To this reviewer, as mentioned in my previous comments, the degradation of proinsulin needs to be measured to determine the contribution of proinsulin degradation to decreased proinsulin content in PDIA6 KD/KO cells.

To address this question, we added a new figure panel **Fig. 5i** and related text in line 340-348.

Proinsulin undergoes both autophagic and proteasomal degradation in β-cells (PMID: 26831301, 35988641). To investigate whether protein degradation contributes to reduced proinsulin levels upon PDIA6 knockdown, we treated cells with two inhibitors—one targeting autophagy (Bafilomycin A1, or BafA1) and the other targeting proteasomal degradation (MG132). If protein degradation was one of the causes, blocking these pathways should have restored proinsulin levels. However, as shown in the new **Figure 5i** shown below, *PDIA6*-depletion lowers proinsulin level regardless of the drug treatment. Therefore, inhibiting autophagy or proteasomal degradation did not rescue proinsulin reduction, further supporting our conclusion that decreased synthesis—not increased degradation—is the primary mechanism following PDIA6 knockdown.

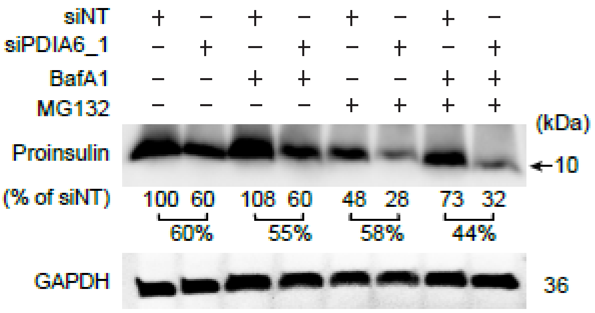

Figure 5i

Also, the total proinsulin (input) in Fig 5h was not significantly decreased. Quantification of newly synthesized proinsulin and steady state proinsulin from same experiments are required to validate the findings.

We thank the reviewer for pointing this out. We have replaced **Fig. 5h** with a new one.

In our previous **Fig. 5h**, the total proinsulin was not significantly decreased, most likely because in that experiment, we performed SUnSET assay only three days after siRNA transfection. At this time, the steady-state proinsulin content was probably still not substantially reduced yet while the proinsulin translation is already low. In the new experiment we performed SUnSET assay 5 days after PDIA6 depletion. As we show in the new **Fig. 5h**, both total and newly synthesized proinsulin are lower after PDIA6 depletion.

2. In the new revised Fig. S3, only one cell was shown in siPDIA6, in which the volume of GM130 appeared to be reduced. Was it a representative cell? Low magnification images may be helpful to make these clearer.

We have included a new **Supplementary Fig. 3a** showing both low-resolution and high-resolution images. Overall, proinsulin co-localizes better with GM130 than with Calnexin except when treating cells with Brefeldin A (an ER-to-Golgi inhibitor). *PDIA6*-depletion does not significantly affect proinsulin location or GM130 signal.

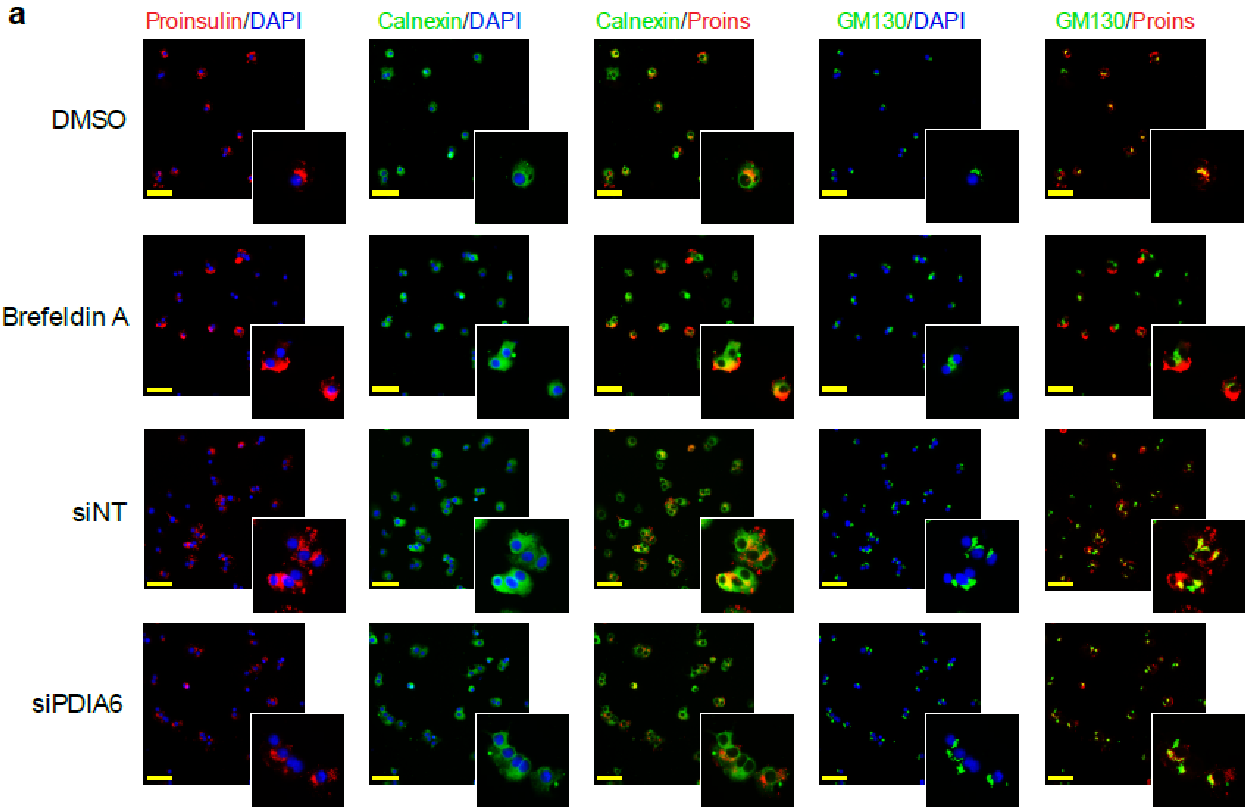

Supplementary Fig. 3a

Reviewer #2 (Remarks to the Author):

The discussion section, though expanded in the revision, is still brief and does not put the new findings in the context of prior work. The discussion section references 7 publications. Three of them (113, 114, 115) refer to insulin clearance work in clinical studies. Two of these refer to prior publications (100,117) about PDIA1. One reference is a clinical study (35). One (51) is a reference to prior publication from this group

Thus, the impact and implications of this work are not clearly stated. How do these findings fit into prior work on proinsulin processing? How does it support or challenge the current model or suggest a new model? For example, on lines 367-375, the manuscript discusses trafficking to and from the Golgi but does not reference prior work or findings or put these findings in the context of what is known about trafficking.

We sincerely thank the reviewer for specific suggestions. We have substantially revised the discussion session (highlighted in yellow) to include more context as the reviewer suggested.

Regarding the context of our proinsulin screen, we cited multiple previous works about the bottleneck of proinsulin processing, which include both exit from ER and exit from Golgi. We note that all previous works drew their conclusions through conventional molecular or cell biology approaches, but our unbiased screens provide a novel genomic perspective which clearly highlights the central role of Golgi in the proinsulin processing. Most of the hits from our screen have not been studied in the context of proinsulin processing.

Regarding PDI and PDIA6, the revision tries to enhance discussion of the significance and novelty of our findings:

- Our study suggests a key role of PDI and PDIA6 in proinsulin processing since PDIA6 is the strongest positive hit.
- The phenotype of PDIA6 perturbation is opposite to the assumption that PDIs function through helping proinsulin folding.
- Our results support PDIA6’s mechanism as a proinsulin translation regulator instead of a player in UPR signaling as suggested in previous publication.

We thank the reviewers for additional comments and have addressed the remaining concerns below. The reviewers' comments are in blue, and our responses are in black.

Reviewer #1 (Remarks to the Author):

Thank authors for their efforts. The manuscript was improved. However, two points related my previous comments need to be addressed:

1. The authors performed inhibitor-based experiments to test whether increased degradation contributes to reduced proinsulin levels upon PDIA6 knockdown. While the finding that MG132 and BafA1 treatment did not restore proinsulin levels provides supportive indirect evidence, this approach does not directly assess proinsulin degradation. To support the conclusion that decreased synthesis—rather than enhanced degradation—is responsible, I suggest including direct measurements of proinsulin degradation kinetics, such as pulse-chase or cycloheximide-chase assays.

We thank the reviewer for the suggestion. To address this question, we performed the cycloheximide (CHX) chase assay using CHX (50 μM) treatment in EndoC-βH3 cells transfected with either siNT or siPDIA6. CHX was applied to block new protein synthesis, and cells were harvested at a series of time points (0, 0.5, 1, 2, 3, 4, and 5 hours) following CHX treatment. Proinsulin levels were assessed by Western blotting with α-tubulin as loading control. Band intensities were quantified and plotted for both transfection groups.

Data from three biological replicates are shown in the new **Figure 6c**. As expected, the starting proinsulin level at 0 h was markedly lower in siPDIA6 cells compared to siNT. The rate of proinsulin decay in siPDIA6 cells, reflected by the percentage of initial proinsulin level over time, was at least not higher than control cells.

Taking together with our previous MG132 and BafA1 experiments (**Figure 5i**), we conclude that PDIA6 knockdown does not accelerate proinsulin degradation.

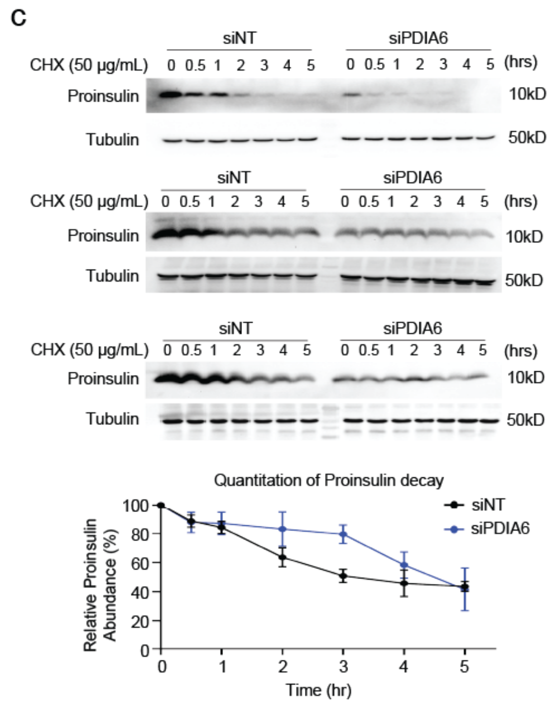

Fig. 6c. CHX chase of proinsulin decay.

2. In the revised Fig. 5h, the inclusion of relative band intensities suggests densitometric quantification was performed. However, as it appears to be based on a single experiment without biological replicates or statistical analysis, the conclusions would be strengthened by including multiple data with appropriate quantification. In addition, it would be helpful to clearly

state the rationale for selecting the altered time point (5 days post-knockdown instead of 3) in the main text or figure legend, to ensure transparency of the experimental design.

We thank the reviewer for the suggestion. The experiment is now performed with three biological replicates, and full quantification with statistical analysis is provided in the revised **Fig. 6b**. Across all replicates, both input proinsulin and IP-proinsulin levels were reduced in siPDIA6 cells.

Regarding the choice of time post-knockdown, we typically perform PDIA6 knockdown in MIN6 cells for 3 days and in EndoC-βH3 cells for 5 days. The reason is that MIN6 cell, as a cell line derived from mouse insulinoma, proliferates faster than EndoC-βH3 cells with doubling time approximately 2–3 days. In contrast, EndoC-βH3 is a human pancreatic β-cell line immortalized by a SV40LT$^{flox/flox}$ transgene. This cell line needs to enter a non-proliferative state (by tamoxifen-induced Cre-ERT2-mediated SV40LT-excision) to adopt a β-cell-like state. Based on prior experiences, we choose to do knockdown experiments in EndoC-βH3 cells for 5-days because we expect slower protein turnover rate when cells are in a non-proliferative state. We have clarified this in the **Methods** section of the revised manuscript (highlighted in yellow).

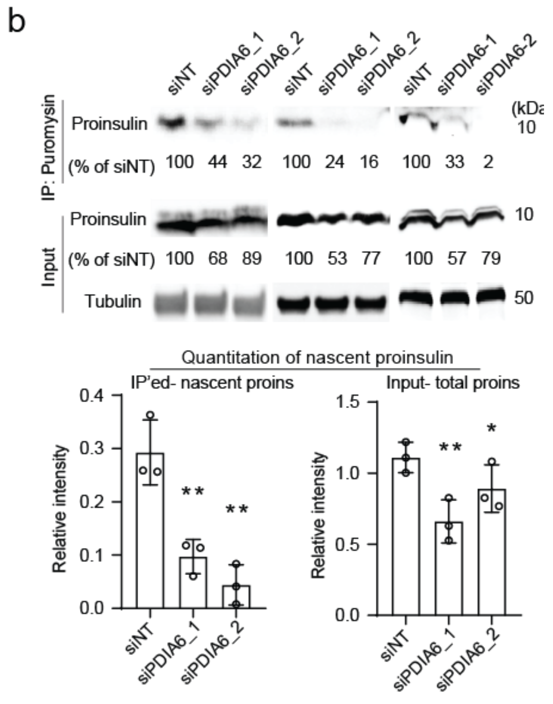

Fig. 6b. Three replicates of SUnSET assays
